# Brain mediators of negative affect-induced physical symptom reporting in patients with functional somatic syndromes

**DOI:** 10.1038/s41398-023-02567-3

**Published:** 2023-08-21

**Authors:** Katleen Bogaerts, Maaike Van Den Houte, Daniëlle Jongen, Huynh Giao Ly, Eline Coppens, Koen Schruers, Ilse Van Diest, Tack Jan, Peter Van Wambeke, Bogdan Petre, Philip A. Kragel, Martin A. Lindquist, Tor D. Wager, Lukas Van Oudenhove, Omer Van den Bergh

**Affiliations:** 1 REVAL - Rehabilitation Research Center, Faculty of Rehabilitation Sciences, Hasselt University, Diepenbeek, Belgium; 2 Health Psychology, Psychology and Educational Sciences, University of Leuven, Leuven, Belgium; 3 Laboratory for Brain-Gut Axis Studies (LaBGAS), Translational Research Center for Gastrointestinal Disorders (TARGID), University of Leuven, Leuven, Belgium; 4 Leuven Brain Institute, University of Leuven, Leuven, Belgium; 5 University Psychiatric Center KU Leuven, University Hospitals Leuven, Leuven, Belgium; 6 MHeNS School for Mental Health and Neuroscience, Maastricht University, Maastricht, The Netherlands; 7 GI motility and sensitivity research group, Translational Research Center for Gastrointestinal Disorders (TARGID), University of Leuven, Leuven, Belgium; 8 Department of Physical and Rehabilitation Medicine, University Hospitals Leuven, Leuven, Belgium; 9 Cognitive & Affective Neuroscience Lab (CANLab), Department of Psychological and Brain Sciences, Dartmouth College, Hanover, NH USA; 10 Department of Psychology, Emory University, Atlanta, GA USA; 11 Biostatistics, Johns Hopkins School of Public Health, Baltimore, MD USA

**Keywords:** Neuroscience, Human behaviour

## Abstract

Functional somatic syndromes (FSS) include fibromyalgia, irritable bowel syndrome (IBS), and others. In FSS patients, merely viewing negative affective pictures can elicit increased physical symptoms. Our aim was to investigate the neural mechanisms underlying such negative affect-induced physical symptoms in FSS patients. Thirty patients with fibromyalgia and/or IBS and 30 healthy controls (all women) watched neutral, positive and negative affective picture blocks during functional MRI scanning and rated negative affect and physical symptoms after every block. We compared brain-wide activation during negative versus neutral picture viewing in FSS patients versus controls using robust general linear model analysis. Further, we compared neurologic pain signature (NPS), stimulus intensity-independent pain signature (SIIPS) and picture-induced negative emotion signature (PINES) responses to the negative versus neutral affect contrast and investigated whether they mediated between-group differences in affective picture-induced physical symptom reporting. More physical symptoms were reported after viewing negative compared to neutral pictures, and this effect was larger in patients than controls (*p* = 0.025). Accordingly, patients showed stronger activation in somatosensory regions during negative versus neutral picture viewing. NPS, but not SIIPS nor PINES, responses were higher in patients than controls during negative versus neutral pictures (*p* = 0.026). These differential NPS responses partially mediated between-group differences in physical symptoms. In conclusion, picture-induced negative affect elicits physical symptoms in FSS patients as a result of activation of somatosensory and nociceptive brain patterns, supporting the idea that affect-driven alterations in processing of somatic signals is a critical mechanism underlying FSS.

## Introduction

Functional somatic syndromes (FSS) such as fibromyalgia and irritable bowel syndrome (IBS) are conditions characterized by debilitating physical symptoms with insufficient identifiable organic cause [1, 2]. FSS are theoretically closely related to Somatic Symptom Disorder (SSD) in DSM-5, but recent studies show that less than 30% of fibromyalgia and IBS patients fulfill the criteria for SSD [3, 4]. The International Association for the Study of Pain (IASP) collectively classifies these disorders as chronic primary pain (CPP) syndromes, agnostic with regard to etiology (to avoid the “physical” versus “psychological” dichotomy), while also allowing for subtypes [5]. The pathophysiological mechanisms underlying symptom generation in FSS indeed remain poorly understood, and no specific diagnostic biomarkers have been identified so far. The current consensus is that FSS are multifactorial, comprising predisposing, precipitating and perpetuating physiological and psychological factors at several levels of functioning. Recent theories point to FSS as disorders of brain-body interaction and give a prominent role to perceptual dysregulation as a mechanism underlying FSS symptoms [6, 7].

One interesting observation is a pervasive correlation between general negative affectivity (NA) and elevated physical symptom reporting. This correlation is found in non-consulting individuals [8], in primary care patients [9], and in secondary care where patients with FSS show higher levels of mental disorders [10, 11]. Acute negative affective states also exacerbate the experience of physical symptoms in healthy individuals and in patients suffering from fibromyalgia and IBS in daily life [12–14]. Interestingly, the mere induction of negative affect through affective picture viewing also induced elevated physical symptom reporting in non-clinical high symptom reporters and in patients with FSS [15–18]. These findings suggest that in individuals with functional somatic symptoms, negative affect co-activates symptom perception processes [18]. However, the neural mechanisms underlying this close connection between somatic (i.e., arising from the entire body, including the viscera) and affective information in FSS are largely unknown.

Studies investigating brain responses to experimentally induced pain or other physical symptoms in FSS patients generally showed stronger patterns of activation in regions related to both somatic/nociceptive and affective processing in FSS patients compared to healthy controls [19, 20]. Studies inducing additional psychosocial stress during rectal distention showed increased activation in the insula, midcingulate cortex, and ventrolateral prefrontal cortex in IBS patients compared to healthy controls [21]. Experimentally induced anticipatory fear before rectal distension caused higher activation of the anterior midcingulate cortex, thalamus, and visual processing areas in IBS patients compared to healthy controls [22], while during distention, higher activation in patients was observed in posterior cingulate and mid-cingulate cortices. Conversely, the use of positive pictures during pain induction caused smaller reductions in pain experience in fibromyalgia patients compared to healthy controls, accompanied by smaller reductions in activation in secondary somatosensory cortex, insula, orbitofrontal cortex, and anterior cingulate cortex [23].

While these studies showed altered patterns of brain activation in response to (noxious) somatic stimulation, it remains unclear how exactly they interact with affective processing. Since pain itself is also an affective experience, somatic and affective stimulation are inherently confounding. Because induction of negative affect alone elicits elevated physical symptoms in patients with FSS compared to healthy controls, the present study using negative picture viewing without somatic stimulation aimed to identify brain mediators underlying the effect of negative affect on physical symptom reporting. Our paradigm has several advantages. First, it allows to disentangle the effects of brain networks involved in negative affect and (noxious) somatic stimulus processing on elevated symptom reporting in FSS. This is relevant because several theoretical accounts of FSS assume a critical role of augmented sensory processing of actual (noxious) somatic stimulation in the brain (e.g., “central sensitization”). Second, brain activation patterns involved in picture-induced negative affect have been thoroughly investigated in healthy subjects, which has resulted in a well-documented neural signature (pattern of brain activation and deactivation) predicting negative emotion ratings in response to negative pictures—the picture-induced negative emotion signature (PINES) [24]. Third, signatures for both the nociceptive/intensity-encoding aspects on non-nociceptive/intensity-independent (e.g., endogenous pain modulatory processes in the brain) of pain have been extensively documented and clearly separated from picture-induced negative affect and other salient aversive events in healthy subjects [25]. The neurologic pain signature (NPS) predicts pain ratings in response to painful stimuli [26] while the stimulus intensity-independent pain signature (SIIPS) predicts pain ratings independent of/beyond stimulus intensity [27].

Hence, the present paradigm allows us to test whether negative affective picture-induced physical symptoms in FSS are mediated through altered nociceptive (NPS), stimulus-independent (SIIPS) and/or negative affective (PINES) brain signature responses, as well as to explore (methods, results, and discussion in Supplementary Materials) potential brain networks mediating this relationship beyond these established signatures. In other words, the question under study is whether patients with FSS report more physical symptoms in response to negative affective stimuli compared to healthy controls because of a relatively stronger activation of pain (NPS, SIIPS) and/or negative affective (PINES) signatures (or other brain networks) in response to these stimuli. In addition, we will also explore (methods, results and discussion in supplement) the role of some psychosocial factors that modulated the effects of negative affect on symptom reporting in previous studies [16, 18]. In sum, our goal was to investigate neural mediators of negative affect-induced physical symptoms in patients with FSS, both in terms of established signatures for nociceptive (NPS) and non-nociceptive (SIIPS) aspects of (evoked) pain as well as picture-induced negative affect (PINES) (hypothesis-driven), and of newly identified (i.e., data-driven) networks.

## Materials and methods

### Participants

All patients were recruited at the University Hospitals Leuven (Leuven, Belgium). Fibromyalgia patients were diagnosed by a physical and rehabilitation medicine specialist (PVW) according to the 1990 ACR criteria for fibromyalgia [28]. IBS patients were diagnosed by a gastroenterologist (JT) according to the ROME III criteria for functional gastrointestinal disorders [29]. Patients were only included if no structural cause for their symptoms could be identified after a systematic medical work-up. Healthy controls were recruited through local advertisement and matched for age and socioeconomic status through frequency sampling. They were only included if they reported low levels of physical symptoms in daily life (i.e., a score <75 on the Checklist for Symptoms in Daily Life) [30]. This cutoff score has shown favorable discriminative power in earlier studies [15]. Only female participants were included to (1) achieve a homogeneous sample not confounded by sex differences which we would not be powered for to detect, and (2) reflect female predominance of FSS. Exclusion criteria for all participants were: currently any history of pulmonary, cardiovascular, gastrointestinal, or neuromuscular chronic illness, or other medical conditions such as acute illnesses or fever, and being pregnant or lactating, and currently receiving psychotherapy. Pharmacological treatment was limitedly allowed and was kept stable from recruitment until two weeks after study participation. Specifically, patients could be included if they were on a stable dose of tramadol, a stable dose of one antidepressant, antihistamines, myorelaxants, or nonopioid analgesics. Additionally, the Dutch version of the MINI International Neuropsychiatric Interview (Version 5.0.0, based on the DSM-IV criteria) was used to exclude participants suffering from any major mental disorders (with the exception of somatization or somatoform disorder for the patient groups) [31, 32].

Thirty-three patients (18 fibromyalgia patients and 15 IBS patients) and 30 controls participated in the study. Data collection took place between August 2012 and November 2018. All participants provided written informed consent before participating in the study. The study was approved by the Social and Societal Ethics Committee of University of Leuven and the Medical Ethical Committee of University Hospitals Leuven. Participants received a financial compensation of 50 euros and reimbursement of traveling costs. A sensitivity analysis (performed with G*Power 9.1.9.7) indicated that our sample size of *n* = 30 per group yields 80% power to detect a medium effect size of Cohen’s *d* = 0.74 for a two-tailed two-sample *t* test.

### Paradigm

#### Stimuli

Seventy-two pictures per valence category (negative, positive, neutral) were selected from the International Affective Picture System [33]. Across valence categories, pictures were equated with each other in terms of luminance, number of close-up faces, number of human figures at a distance, number of animals, and number of simple objects versus complex scenes. Within valence blocks, pictures were equated using norms for valence and arousal and negative pictures had similar fear and sadness ratings, using normative data collected by Mikels et al. [34]. Only low disgust pictures were included in the stimulus set [34]. For more information about the used affective picture paradigm and selected pictures, we refer to Bogaerts et al. [16].

#### Behavioral measures

*State negative affect* was measured using the negative affect subscale of the state version of the Positive and Negative Affect Schedule (PANAS; [35]). Participants indicated to what extent they were experiencing each of ten listed negative emotions at that moment on a numeric ratings scale from 1 (not at all) to 5 (very much). For the purpose of the study, i.e., to avoid too many ratings after each block, the scale was divided into two subsets based on item content (subset 1: distressed, nervous, hostile, guilty, scared; subset 2: upset, jittery, irritable, ashamed, afraid). *State physical symptoms* were measured with a symptom checklist rating the current experience of ten physical symptoms on a numeric ratings scale from 1 (not at all) to 5 (very much). The symptom checklist was divided in 2 subsets as well (subset 1: tightness of the chest, heart pounding, stomach/abdominal pain, headache, fatigue; subset 2: not being able to breathe deeply enough, rapid heartbeat, nausea, dizziness, muscle pain). This symptom checklist has been used previously in similar studies in our lab [16, 18].

#### Design

**Fig. 1:**
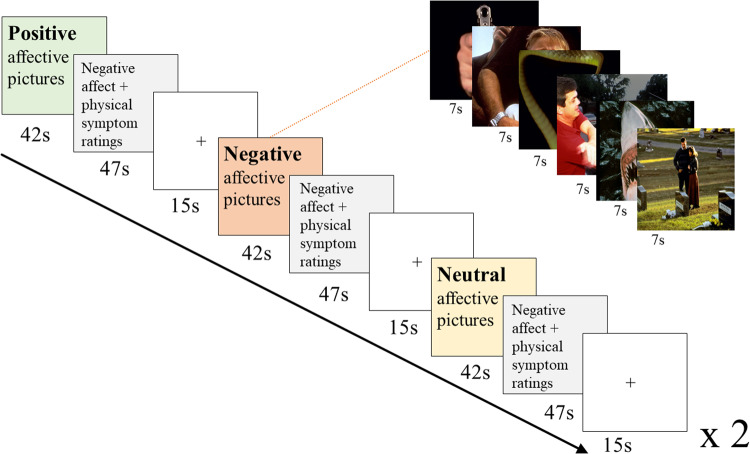
Schematic depiction of one of the six runs of the picture viewing paradigm. As indicated by the “×2,” series of the three picture valences were presented twice in one run.

Participants went through six runs of picture viewing. Between each of the six runs there was a one minute pause. Each run consisted of six blocks with different picture valences (2 negative, 2 positive, and 2 neutral blocks), with negative affect and physical symptom ratings after each block (Fig. 1). See Supplementary Materials for detailed description.

### fMRI methods

Data were acquired on a 3 T Philips Achieva DStream MRI scanner with a 32-channel head coil (Philips Medical Systems, Best, The Netherlands). See Supplementary Materials for detailed description of fMRI data acquisition, preprocessing, and quality control.

### Statistical analysis

#### Behavioral data analysis

Statistical Analysis Software (SAS) version 9.4 (TS1M6) (SAS Institute, Cary, NC, USA) was used for all non-brain imaging data analyses. Behavioral data were analyzed at the subject level. Physical symptom and negative affect items from within one run were added up and averaged over runs, leading to both a physical symptom score and a negative affect score ranging from 10 to 50 for each of the three picture valences.

Due to skewness of both the negative affect and physical symptom ratings and the impossibility to normalize the dependent variable through transformation, generalized estimating equation (GEE) models were used (SAS proc genmod) to fit normal, gamma, and negative binomial distributions. The latter were shown to fit the data best based on the lowest value of Akaike’s Information Criterion (AIC). *“Group”* (controls versus FSS patients, between-subject) and “*affective valence*” (negative, neutral, positive; within-subject) were included as independent variables in the model, including both main effects and their interaction. Following up on the group × affective valence interaction effect, between-group differences for the planned contrasts “negative versus neutral” and “negative versus positive” were tested using two unpaired *t* tests on the GEE model estimates, with stepdown Bonferroni (Holm) multiple testing correction. This tests our hypothesis of increased physical symptom reporting after negative compared to both neutral and positive pictures in FSS patients compared to controls.

#### fMRI data analysis

We used the neuroimaging analysis tools developed by the Cognitive and Affective Neuroscience Lab (CANlab) at Dartmouth College (https://github.com/canlab). See Supplementary Materials for details on first-level analysis.

##### Second-level analysis

To test our hypothesis of increased brain response to negative versus neutral and positive affective pictures in patients versus controls, we performed the following analyses on the first-level contrasts negative versus neutral and negative versus positive (based on behavioral findings that showed no difference in symptom reporting between positive and neutral affective pictures, we did not investigate the neutral versus positive contrast [15–18]:

1. a whole-brain parcel-wise robust general linear model analysis comparing patients versus controls in each of the 489 brain parcels of the CANlab 2018 combined atlas (https://sites.google.com/dartmouth.edu/canlab-brainpatterns/brain-atlases-and-parcellations/2018-combined-atlas) while controlling for the respective contrast in physical symptom ratings, thresholded at whole-brain *q*_FDR_ < 0.05. Briefly, this analysis compares the average response in each of the parcels between groups, while correcting for the number of tests/parcels. See Supplementary Materials for detailed information about the atlas.
2. comparing the NPS, SIIPS, and PINES response (calculated using the dot product metric, which is a voxel-wise multiplication of the beta weight in the contrast image and the beta weight in the pattern map) to these contrasts between patients and controls by independent samples *t* test, followed up by breaking up the results by subregions of these patterns in case of significance for the entire pattern.

To study brain mechanisms underlying the between-group differences in negative affect-induced physical symptoms, we performed (single-level) mediation analysis with the independent variable X being “group” (FSS patients versus controls), the dependent variable Y being the difference in physical symptom rating for the negative versus neutral affective valence contrast, and the mediator M being the brain response to the respective contrast. Two complementary mediation analyses were performed using the CANlab mediation toolbox (https://github.com/canlab/MediationToolbox) [36]. More specifically, the brain response was used as a mediator in the following two complementary ways:

1. univariate mediation analysis with the NPS response as the mediator, i.e., Group → NPS → physical symptoms.
2. whole-brain multivariate mediation analysis using the “principal directions of mediation” (PDM) method [37, 38].

See Supplementary Materials for detailed information about the mediation analyses.

## Results

### Participant characteristics

The final sample consisted of 30 patients (15 fibromyalgia patients, 15 IBS patients; mean age = 42.91 SD = 11.34; all women) and 30 controls (mean age = 43.0, SD = 11.81; all women). Six of the IBS patients also fulfilled the 2011 ACR criteria for fibromyalgia [28] based on a self-report checklist. See Table 1 for information on education level and medication use.

**Table 1:** Demographic information.

	FSS patients		Controls	
	Mean	SD	Mean	SD
Age	43.3	11.34	42.97	11.81
Educational level				
High school	44.44%		16.67%	
College	40.74%		36.67%	
University	14.81%		46.67%	
Medication use				
Tramadol (opioid)	23.33%		0%	
Non-opioid analgesics	26.67%		0%	
Antidepressants	50%		0%	
At least one of the above	76.67%		0%	

For two participants (1 control, 1 patient), no behavioral ratings were collected during scanning due to equipment malfunction. They were excluded in all analyses of behavioral data but retained in other analyses. More details on fMRI data quality control and final sample can be found in Supplementary Materials.

### Behavioral results

#### Negative affect (Fig. 2a)

Negative affect ratings were higher in patients versus controls, regardless of affective picture valence (main effect of Group [*χ*^2^(1) = 17.51, *p* < 0.0001, least square means (LSM) estimates 2.49 ± 0.019 versus 2.66 ± 0.036]). Negative affect ratings were higher after negative versus both positive and neutral valence pictures, across both groups (main effect of Affective Valence [*χ*^2^(2) = 58.95, *p* < 0.0001]). Post hoc tests showed a significant difference between negative valence pictures on the one hand (LSM estimate 2.83 ± 0.047) and both neutral (LSM estimate 2.45 ± 0.023, *p*_Holm_ < 0.0001) and positive (LSM estimate 2.44 ± 0.032, *p*_Holm_ < 0.0001) pictures. The Affective Valence × Group interaction effect was non-significant [*χ*^2^(2) = 0.28, *p* = 0.87].

**Fig. 2:**
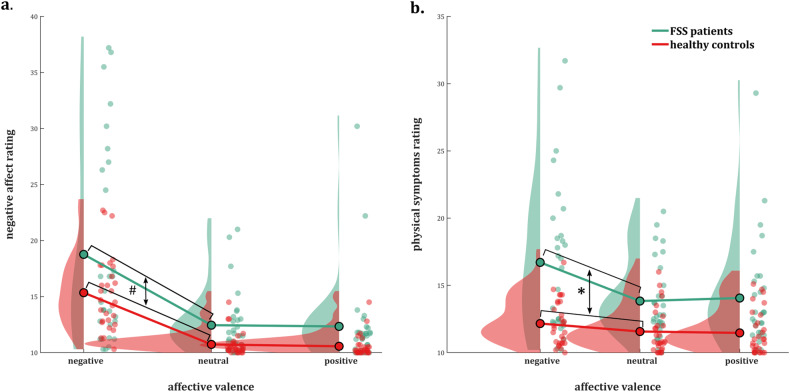
Negative affect and somatic symptom ratings. State negative affect (**a**) and physical symptom (**b**) ratings in functional somatic syndrome patients and healthy controls, by picture valence. Planned contrasts indicated that the difference in physical symptom ratings between negative and neutral pictures was larger in patients compared to controls (LSMdiff estimate 0.19 ± 0.053 versus 0.05 ± 0.015). For negative affect ratings, this contrast was not significant. *pHolm = 0.025, ^#^pHolm > 0.99.

#### Physical symptoms (Fig. 2b)

Patients had higher symptom ratings than controls, regardless of affective valence (main effect of Group [*χ*^2^(1) = 26.05, *p* < 0.0001, LSM estimates 2.70 ± 0.038 versus 2.46 ± 0.025]. Physical symptom ratings were higher after negative versus both positive and neutral valence pictures, across both groups (main effect of Affective Valence [*χ*^2^(2) = 19.35, *p* < 0.0001]). Post hoc tests showed a significant difference between negative valence pictures (LSM estimate 2.66 ± 0.033) and both neutral (LSM estimate 2.54 ± 0.023, *p*_Holm_ < 0.0001) and positive (LSM estimate 2.54 ± 0.030, *p*_Holm_ = 0.006) pictures. Importantly, the difference in physical symptom ratings between valences differed between patients versus controls (Affective Valence × Group interaction effect: *χ*^2^(2) = 7.30, *p* = 0.026). Planned contrasts indicated that the difference in physical symptom ratings between negative and neutral pictures was larger in patients compared to controls (LSM_diff_ estimate 0.19 ± 0.053 versus 0.05 ± 0.015, *p*_Holm_ = 0.025). The difference between physical symptom ratings in the negative versus positive condition did not differ significantly between patients and controls (LSM_diff_ estimate 0.17 ± 0.078 versus 0.06 ± 0.013, *p*_Holm_ = 0.15).

### fMRI results

The results below refer to our primary contrast of interest, i.e., negative versus neutral contrast. See Supplementary Materials for results on the negative versus positive contrast.

#### Whole-brain parcel-wise analysis

**Fig. 3:**
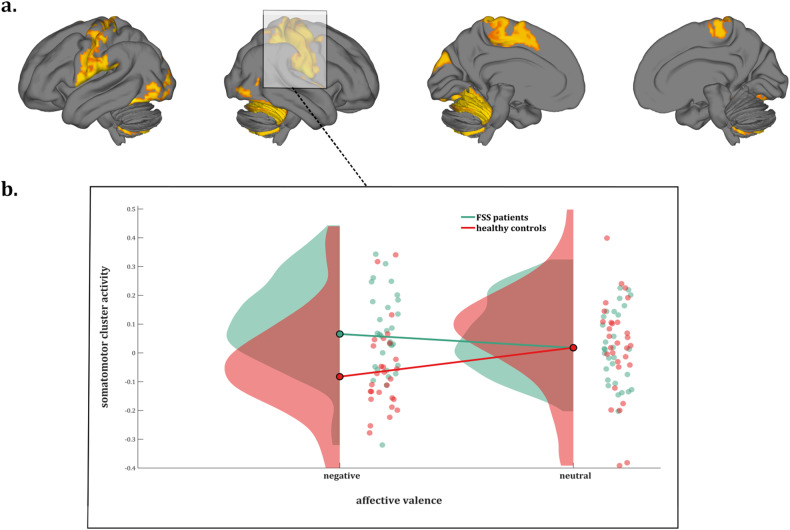
Whole-brain parcel-wise analysis. **a** Whole brain parcel-wise GLM results, for the contrast negative > neutral valence in functional somatic syndrome patients vs. healthy controls and **b** response in the somatomotor cluster in patients and controls during negative and neutral picture viewing.

For the negative > neutral contrast, a significantly stronger response was found in patients versus controls in somatosensory/motor regions [including posterior insula, opercular areas/secondary somatosensory cortex (SII), primary somatosensory cortex (SI), primary motor cortex (M1), supplementary motor area (SMA), and anterior midcingulate cortex (aMCC), as well as cerebellar and (higher order) visual cortical regions (Fig. 3, Supplementary Fig. S2, and Supplementary Table S2).

No areas showed stronger responses in controls versus patients.

#### Signature responses

##### Neurologic Pain Signature (NPS)

**Fig. 4:**
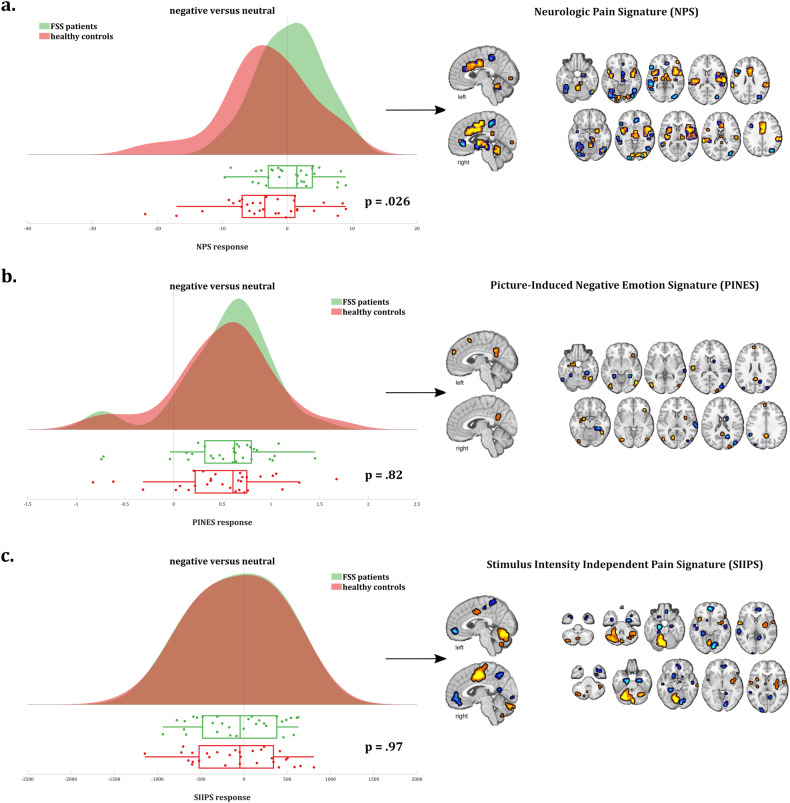
Signature responses. **a** NPS response, **b** PINES response, and **c** SIIPS response in functional somatic syndrome patients vs. healthy controls for the negative > neutral valence contrast, including visual representation of the three neural signatures.

Entire pattern: For the negative > neutral contrast, a significant though weak negative NPS response (i.e., lower pattern expression in the negative compared to the neutral valence category) was found in controls (dot product −3.07 ± 1.28, *t*(29) = −2.40, *p* = 0.023, Cohen’s *d* = −0.44), which was absent in patients (dot product 0.50 ± 0.88, *t*(29) = 0.57, *p* = 0.57, Cohen’s *d* = 0.10), resulting in a significant between-group difference [*t*(51.3) = 2.30, *p* = 0.026] (Fig. 4a). The NPS responses to the negative and neutral conditions for patients and controls are summarized in Supplementary Table S3.

To further interpret these results (in terms of which subpatterns/regions within the NPS are driving them), we broke the entire NPS pattern response down in (sets of) regions constituting the NPS.

**Fig. 5:**
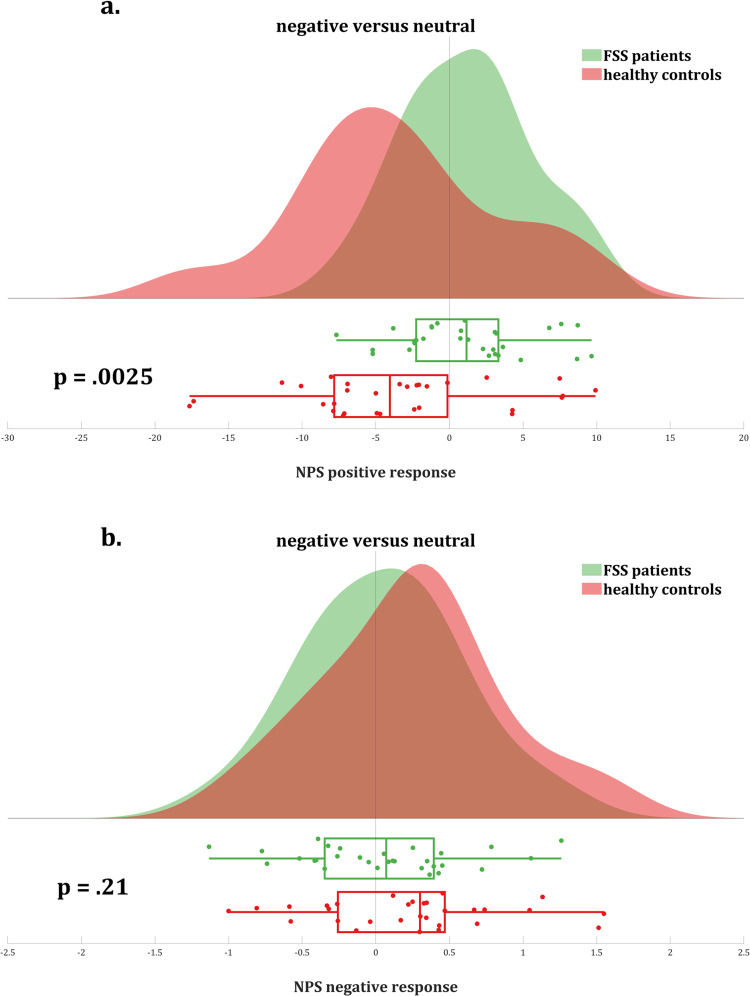
NPS response subpatterns and subregions. Brain response to negative > neutral valence in the NPS subpatterns with **a** positive and **b** negative weights in functional somatic syndrome patients vs. healthy controls.

Subpatterns and subregions: The NPS consists of regions with positive (i.e., higher neural response predicting higher pain ratings) and negative (i.e., lower neural response predicting higher pain ratings) predictive weights. Hence, we first split the pattern in two subpatterns accordingly: one pattern including all NPS regions with positive weights (“positive NPS subpattern”), the other one including all NPS regions with negative weights (“negative NPS subpattern”). Results show that the abovementioned results for the NPS as a whole are driven by the positive NPS subpattern (see below for details of regions). More specifically, for the negative > neutral contrast, the significant between-group difference was more pronounced in the positive NPS subpattern compared to the negative NPS subpattern [*t*(49.4) = 3.18, *p* = 0.0025], with controls showing a significant negative response of medium size (dot product −3.48 ± 1.25, t(29) = −2.78, *p* = 0.0095, *d* = -0.51) and patients showing a small but non-significant positive response (dot product 1.26 ± 0.80, *t*(29) = 1.57, *p* = 0.13, *d* = 0.29) in the positive NPS subpattern (Fig. 5a).

No significant between-group effects were found for the negative NPS subpattern, nor was a significant negative versus neutral difference observed within any of both groups. However, numerically stronger responses were found in controls compared to FSS patients (*p* = 0.21; Fig. 5b).

**Fig. 6:**
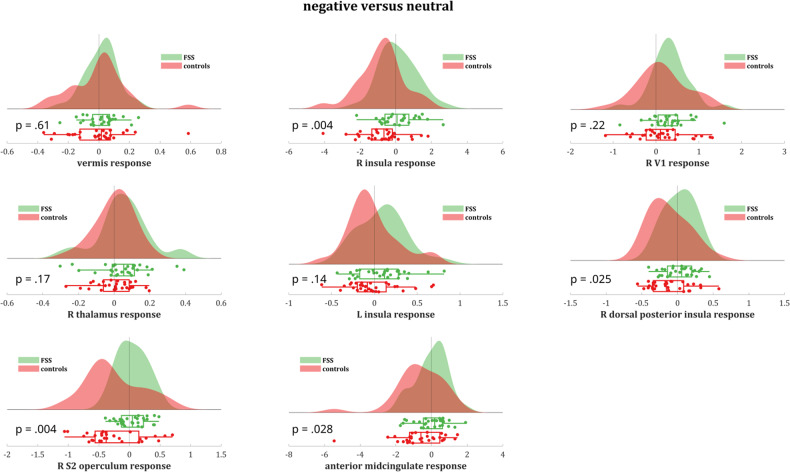
Brain response to negative > neutral valence in key individual regions of the NPS subpattern with positive weights in functional somatic syndrome patients versus healthy controls. *p* Values are false discovery rate (FDR)-corrected for multiple comparisons.

To further clarify the results in the positive NPS subpattern with positive predictive weights for the negative > neutral contrast, and test for regional specificity, we explored between-group differences in 8 key regions of the pattern. Significantly stronger activations (negative > neutral) were found in patients compared to controls in the right insula (qFDR = 0.004), right dorsal posterior insula (qFDR = 0.025), right S2/operculum (qFDR = 0.0041), and aMCC (qFDR = 0.028), but not in cerebellar vermis, right V1, right thalamus, or left insula (qFDR = 0.61, 0.22, 0.17, and 0.14, respectively) (Fig. 6).

##### Picture-induced Negative Emotion Signature (PINES)

For the negative > neutral contrast, strong and significant PINES activation was found in both patients (dot product 0.54 ± 0.09, *t*(29) = 6.23, *p* < 0.0001, Cohen’s *d* = 1.14) and controls (dot product 0.51 ± 0.10, *t*(29) = 5.29, *p* < 0.0001, *d* = 0.97), without a significant between-group difference [*t*(57.4) = 0.23, *p* = 0.82] (Fig. 4b). The PINES responses to the negative and neutral conditions for patients and controls are summarized in Supplementary Table S3.

##### Stimulus-intensity Independent Pain Signature (SIIPS)

For the negative > neutral contrast, no significant SIIPS response was found in patients (dot product −64.36 ± 85.95, *t*(29) = −0.75, *p* = 0.46, *d* = −0.14), nor in controls (dot product −68.57 ± 90.05, *t*(29) = −0.76, *p* = 0.45, *d* = −0.14), resulting in a non-significant between-group difference [*t*(57.9) = 0.03, *p* = 0.97] (Fig. 4c). The SIIPS responses to the negative and neutral conditions for patients and controls are summarized in Supplementary Table S3.

#### Mediation analyses

##### Mediation by the NPS response

**Fig. 7:**
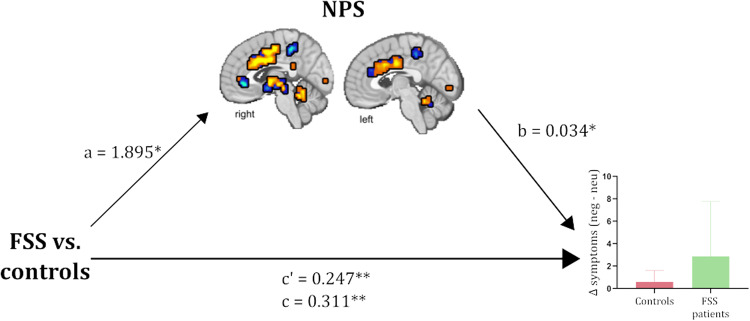
Mediation of the relationship between patient status [functional somatic syndrome (FSS) patient versus healthy control] and physical symptom ratings after negative versus neutral picture viewing by the NPS response. Asterisks indicate pathways significantly different from 0 at *p < 0.05 and **p < 0.01.

As shown in Fig. 7, patients reported more negative affect-induced physical symptoms than controls (path c [total X–Y relationship]: *β* = 0.311 ± 0.124, *p* = 0.002); however, the NPS response also independently predicted physical symptoms (path b: [*β* = 0.034 ± 0.019, *p* = 0.018]), and patients had higher NPS responses versus controls (path a: [*β* = 1.895 ± 0.791, *p* = 0.011]). The indirect effect of Group on negative affect-induced physical symptoms attributable to the NPS response was statistically significant (path a–b (mediated X–Y relationship by M): [*β* = 0.064 ± 0.048, *p* = 0.020]), thus the NPS response mediates group differences in negative affect-induced physical symptoms. The mediation was only partial however, since the direct effect of Group on negative affect-induced physical symptoms remained significant even after controlling for the NPS response (path c′ (residual unmediated X–Y relationship): [*β* = 0.247 ± 0.113, *p* = 0.007]).

##### Principal directions of mediation (PDM)

We identified two independent brain activation patterns mediating the relationship between patient status (patient versus HC) and negative-affect induced symptom reporting using the multivariate PDM analysis method. See Supplementary Materials for detailed results.

## Discussion

The goal of this study was to investigate brain activation patterns underlying negative affect-induced physical symptoms in FSS patients. First, behaviorally we replicated that viewing negative affective pictures elicited elevated physical symptoms in patients compared to controls [15–18]. While patients experienced overall more negative affect throughout picture viewing, this was independent of the affective valence of the pictures. These findings are an exact replication of our earlier studies employing this paradigm. Interestingly, the current study suggests that this effect could also be observed in FSS patients without any mental disorders, as mental disorders were an exclusion criterion for participating in the current study, but not in Van Den Houte et al. [18]. These self-reported findings were corroborated by the imaging data in that there was a stronger activation of somatosensory/motor networks, such as the posterior insula, S1, S2, M1, and aMCC in patients versus controls during negative versus neutral picture viewing. These regions are typically involved in processing of (noxious) somatic stimulation and are part of the so-called “pain matrix” [39].

We also tested whether patients and controls showed different NPS, SIIPS, and PINES responses to negative affective versus neutral pictures, since these signatures can differentiate between neural responses to affective stimuli and nociceptive and non-nociceptive aspects of physical pain [24, 26, 27]. In accordance with the self-reported data, we found relatively stronger negative affect-induced activation of the NPS—but not SIIPS nor PINES—in patients than controls. Moreover, these differences in NPS activation partially mediated the group difference in physical symptoms after viewing negative compared to neutral pictures.

Interestingly, for the same negative > neutral picture valence contrast, no group differences appeared in negative affect nor in activation of its associated neural signature, the PINES. This demonstrates that the stronger somatosensory/nociceptive brain response in FSS patients during negative picture viewing is not due to stronger affect induction, at the self-report nor neural level. In addition, previous studies with this paradigm measuring autonomic arousal (heart rate, electrodermal response) during picture viewing did not show differential arousal effects [13, 15, 16]. Apparently, the brain of FSS patients processes negative affective states as noxious somatic stimulation. This interpretation is further corroborated by our finding that the NPS, but not the SIIPS, was differentially activated in patients versus controls (and mediates the increased symptom response). This implies that sensory/nociceptive rather than extra-nociceptive (including pain modulatory) endogenous cerebral processes mediate negative affect-induced physical symptom reporting in FSS patients.

In addition to the NPS, we also identified two independent brain activation patterns mediating the relationship between patient status (patient versus HC) and negative-affect induced symptom reporting using the multivariate PDM analysis method: (1) a “mediator” pattern, showing stronger negative-affect induced affect activation of regions known to be involved in (noxious) somatic stimulus processing [39] in patients and (2) a “suppressor” pattern, in which patients demonstrated stronger negative affect-induced activation of, among others, regions involved in affect and pain modulation, where stronger activation was related to less physical symptom reporting; and less strong activation of the PAG area (known to be involved in descending pain modulation [40]), cerebellar subregions, and right anterior insula (a higher order area where interoceptive and affective input is integrated to shape conscious experience [41]), with less strong activation being related to higher negative-affect induced symptom reporting. We refer to Supplementary Materials for a thorough discussion of these PDM analyses.

To our knowledge, this is the first brain imaging study showing that mere induction of negative affect without concomitant somatic stimulation induces elevated physical symptoms in patients with fibromyalgia and IBS compared to healthy controls. Previous studies found enhanced activation of pain- and affect-related regions as well as the NPS in response to experimental noxious stimulation in fibromyalgia and IBS patients compared to controls [19, 20, 42]. In this study, however, we demonstrated that similar differences in brain activation patterns occur after mere negative affect induction in the absence of somatic stimulation, and that these responses mediate group differences in self-reported physical symptoms after viewing negative affective pictures. These findings are in line with recent accounts of symptom perception proposing that (noxious) somatic input is not a necessary prerequisite for the experience of physical symptoms [6], but add novel evidence on the underlying brain mechanisms. In that account, symptoms are thought to arise as the end product of a hierarchical inferential process in the brain, involving two counter-flowing streams of information, namely afferent input on the one hand and predictions from the brain on the other hand. The relative contributions of the somatic input and the predictions are determined by their respective precisions (or statistical confidence) [6, 43]. A symptom perception account of FSS assumes reduced detail in somatosensory processing, leaving more room for prediction-based contextual information to determine the eventual somatic percept. The short symptom questionnaire that was presented after each picture viewing block throughout the experiment may have provided the context that stimulated somatic predictions at an early phase of information processing. This is suggested by the abovementioned finding that differential responses to negative affective pictures emerge in (early) visual cortex and mediate increased negative affect-induced physical symptoms in patients versus controls. Furthermore, key regions that have been shown to be involved in aversive prediction error generation [44], such as the vmPFC and (extended) hippocampus (encoding expected value signals) as well as the aMCC, dmPFC (avoidance value updating) and PAG (aversive prediction error encoding) were identified as mediators of the between-group differences in negative affect-induced physical symptoms in our PDM analysis.

There are a number of limitations to our study that need to be addressed. First, we did not investigate differences between patients with fibromyalgia and IBS. Although the different FSS are often lumped together based on common hypothesized pathophysiological mechanisms and high comorbidities amongst the different diagnoses - see also the unifying concept of Bodily Distress Syndrome (BDS, [7, 42]), and commonly classified as CPP by IASP, there is a clear distinction between the two disorders in terms of symptomatology. However, given the relatively small sample size, the fact that almost half of the included IBS patients also reported symptoms of fibromyalgia, and the fact that they are likely similar as to mechanisms involved in symptom perception [7], we focused on the difference between controls and the patient group as a whole. Moreover, from a generalization perspective, our finding that the observed mechanisms generalize across diagnostic FSS categories could be considered a strength. Second, we used mediation as a statistical method, which does not allow us to make strong claims about causality, particularly given the cross-sectional rather than longitudinal nature of the data. Third, the fact that the majority (76%) of the included patients were on medication could be considered a limitation, especially in the light of recent findings that the NPS is responsive to analgesic drug effects on acute evoked pain perception in osteoarthritis patients (which is to be expected for a nociceptive pain signature) [43]. However, analgesics dampened both subjective and NPS responses in this study, hence medication effects are unlikely to explain the relatively *increased* NPS response in patients versus controls in the present study. Moreover, interrupting intake of stable doses of medication in patients for study purposes is hardly feasible (nor indeed ethical), and may cause confounding rebound effects. Given the fact that 76% of the patients and 0% of the controls were on medication, statistical control for medication intake was not possible due to power and multicollinearity issues. Finally, given the female predominance in FSS and for the sake of homogeneity only women were included in this study, so the results are not necessarily generalizable to male FSS patients.

Apart from the mechanistic implications, our findings are also clinically important as they suggest two main treatment strategies for FSS [6, 7]. On the one hand, approaches targeting perceptual differentiation of interoceptive sensations, in particular between emotion and nociception, should be considered to become part of standard care. Increasing sensory-perceptual detail is expected to give more weight to differentiated somatic input and reduce the impact of somatic predictions. On the other hand, approaches weakening chronic somatic predictions as involved in chronic somatic concerns and somatic hypervigilance should further reduce the probability of misconstruing affective states as somatic sensations. Interoceptive exposure may contribute to both strategies [44].

In summary, the present study investigated the brain mechanisms underlying elevated physical symptom reporting in FSS patients after induction of negative affect through picture viewing without any somatic stimulation. Our findings confirmed that picture-induced negative affect elicits physical symptom reports in FSS patients as a result of activation of somatosensory and nociceptive, and, to a lesser extent, pain modulatory brain patterns. These findings support the idea that affect-driven alterations in processing of somatic signals is a critical mechanism underlying FSS.

### Supplementary information

Supplementary materials

## Data Availability

All anonymized data and scripts will be made available on https://github.com/labgas/proj-emosymp.
