# Supplementary material for: Brain mediators of negative affect-induced physical symptom reporting in patients with functional somatic syndromes
Source: Transl Psychiatry. 2023 Aug 21;13:285. doi: 10.1038/s41398-023-02567-3 (PMC10442365; doi:10.1038/s41398-023-02567-3)
Supplement: Supplementary file 1 — Supplementary materials [file 41398_2023_2567_MOESM1_ESM.pdf]

Supplementary Materials

MATERIALS AND METHODS

Paradigm

Design

Participants went through six runs of picture viewing. Between each of the six runs there was a one minute pause. Each run consisted of six blocks with different picture valences (2 negative, 2 positive, and 2 neutral blocks), with negative affect and physical symptom ratings after each block. One block consisted of a picture viewing phase in which participants viewed six different pictures (7s per picture, 42s per block) of the same valence *(Fig. 7)*. Six different orders of block presentation within one run were created (i.e. the six possible orders of the three valences, repeated twice, e.g. pos – neu – neg – pos – neu – neg). Each run had a different order of block presentation, so that every participant received every order of block presentation once throughout the experiment. Consequently, six different orders of runs were created and counterbalanced across participants. One of the subsets (randomly selected) of the questions for both negative affect and physical symptoms was presented after the first three blocks within a run; the other subsets were presented after the last three blocks, so that both the PANAS-negative and the full physical symptom checklist were administered for all valences within one run. The participants had 4s time to answer each item. Stimulus presentation and subjective rating assessment was done using Affect 4.0 software (S1).

Trait questionnaires

For explorative reasons, we also investigated factors that previously have been shown to modulate the effects of negative affect on symptom reporting.

Habitual physical symptom reporting (physical symptom reporting in daily life) was measured using the Checklist for Symptoms in Daily life (CSD) (36). Participants indicate for each of 39 bodily symptoms from various modalities how often they have experienced them in the past year on a 5-point scale (1: never – 5: very often), leading to a score ranging between 39 – 195. Habitual symptom reporting appeared to impact the effects of negative picture viewing on symptom reporting (13-15).

Childhood trauma was measured using the Childhood Trauma Questionnaire (CTQ) (S2). Participants indicate to what extent each of 25 statements regarding their childhood apply to them on a 5 point-scale (1: never true – 5: often true). The questionnaire assesses sexual abuse, physical abuse, emotional abuse, physical neglect, and emotional neglect. Only the total score, ranging between 25 – 125, of the CTQ was used in this study.

Difficulty in Identifying Feelings (DIF) was assessed with the DIF-subscale of the Toronto Alexithymia Scale (S3). The DIF-subscale assesses difficulty identifying and differentiating emotions by having participants rate to what extent they agree with 7 statements on a 5-point scale (1: completely disagree – 5 completely agree), and scores range between 7 and 35. DIF appeared to mediate the effects of negative picture viewing on symptom reporting (14,16).

The *tendency to become absorbed in experiences* was measured with the Tellegen Absorption Scale (S4). Respondents indicate for 39 statements whether it applies (1) or doesn't apply (0) to them, leading to a score ranging between 0 and 39. Absorption appeared to impact the effects of negative picture viewing on symptom reporting (14,16).

fMRI methods

fMRI data acquisition

Anatomical data acquisition

A T1-weighted Turbo Field Echo (TFE) sequence was used, with the following parameters: TR 9.6 ms, TE 4.6 ms, flip angle 8°, coronal slice thickness 1.2 mm, voxel size 0.98 x 1.20 x 0.98mm.

Functional data acquisition

For the purpose of functional data acquisition, 212 T2*-weighted volumes were acquired per run using a gradient echo-planar imaging (EPI) sequence with the following parameters: TR 3000 ms, TE 30 ms, flip angle 90°, 50 transverse slices (ascending order, slice thickness 2.5 mm, slice gap 0.25 mm), voxel size 2.5 x 2.5 x 2.5 mm, parallel imaging, SENSE.

fMRI data preprocessing & quality control

Source data from the scanner were converted from Philips' native PAR/REC format into Nifti using dicm2nii Matlab code (https://github.com/xiangruili/dicm2nii), and data were organized according to the Brain Imaging Data Structure (BIDS) specification (https://bids.neuroimaging.io/) prior to preprocessing.

Mriqc version 0.16.0 (https://mriqc.readthedocs.io/en/stable/) was used for quality control purposes. The following quality criteria were used to exclude a run: (a) >15% of the volumes identified as spikes based on the FD and DVARS thresholds set in fMRIprep (see below), and (b) AFNI quality index (aqi) > 0.02. The aqi is a global quality index provided as part of the mriqc output (https://afni.nimh.nih.gov/pub/dist/doc/program_help/3dTqual.html).

Preprocessing was performed using *fMRIPrep* 20.2.1 (RRID:SCR_016216) (S5,S6), which is based on *Nipype* 1.5.1 (RRID:SCR_002502) (S7). Many internal operations of *fMRIPrep* use *Nilearn* 0.6.2 (RRID:SCR_001362 (S8), mostly within the functional processing workflow. The fMRIPrep pipeline uses a combination of tools from well-known software packages, including FSL, ANTs, FreeSurfer and AFNI. This pipeline was designed to provide the best software

implementation for each state of preprocessing and will be updated as newer and better neuroimaging software become available. For more details of the pipeline, see https://fmriprep.org/en/latest/workflows.html.

Anatomical data preprocessing

The T1-weighted (T1w) image was corrected for intensity non-uniformity (INU) with N4BiasFieldCorrection (S9), distributed with ANTs 2.3.3 (RRID:SCR_004757) (S10), and used as T1w-reference throughout the workflow. The T1w-reference was then skull-stripped with a *Nipype* implementation of the antsBrainExtraction.sh workflow (from ANTs), using OASIS30ANTs as target template. Brain tissue segmentation of cerebrospinal fluid (CSF), white-matter (WM) and gray-matter (GM) was performed on the brain-extracted T1w using fast (FSL 5.0.9, RRID:SCR_002823) (S11). Volume-based spatial normalization to one standard space (MNI152NLin2009cAsym) was performed through nonlinear registration with antsRegistration (ANTs 2.3.3), using brain-extracted versions of both T1w reference and the T1w template. The following template was selected for spatial normalization: *ICBM 152 Nonlinear Asymmetrical template version 2009c* (RRID:SCR_008796; TemplateFlow ID: MNI152NLin2009cAsym) (S12).

Functional data preprocessing

For each of the 6 BOLD runs found per subject (across all tasks and sessions), the following preprocessing was performed. First, a reference volume and its skull-stripped version were generated using a custom methodology of *fMRIPrep*. Susceptibility distortion correction (SDC) was omitted. The BOLD reference was then co-registered to the T1w reference using flirt (FSL 5.0.9) with the boundary-based registration cost-function (S13,S14). Co-registration was configured with nine degrees of freedom to account for distortions remaining in the BOLD reference. Head-motion parameters with respect to the BOLD reference (transformation matrices, and six corresponding rotation and translation parameters) are estimated before any spatiotemporal filtering using mcflirt (S15). BOLD runs were slice-time corrected using 3dTshift from AFNI 20160207 (RRID:SCR_005927) (S16). The BOLD time-series (including slice-timing correction when applied) were resampled onto their original, native space by applying the transforms to correct for head-motion. These resampled BOLD time-series will be referred to as *preprocessed BOLD in original space*, or just *preprocessed BOLD*. The BOLD time-series were resampled into standard space, generating a *preprocessed BOLD run in MNI152NLin2009cAsym space*. First, a reference volume and its skull-stripped version were generated using a custom methodology of *fMRIPrep*. Several confounding time-series were calculated based on the *preprocessed BOLD*: framewise displacement (FD), DVARS and three region-wise global signals. FD was computed using two formulations following Power (absolute sum of relative motions) and Jenkinson (relative root mean square displacement between affines) (S15,S17). FD and DVARS were calculated for each functional run, both using their implementations in *Nipype* (following the definitions by Power et al. (S17). The three global signals were extracted within the CSF, the WM, and the whole-

brain masks. Additionally, a set of physiological regressors were extracted to allow for component-based noise correction (*CompCor)* (S18). Principal components were estimated after high-pass filtering the *preprocessed BOLD* time-series (using a discrete cosine filter with 128s cut-off) for the two *CompCor* variants: temporal (tCompCor) and anatomical (aCompCor). tCompCor components are then calculated from the top 2% variable voxels within the brain mask. For aCompCor, three probabilistic masks (CSF, WM and combined CSF+WM) are generated in anatomical space. The implementation differs from that of Behzadi et al. in that instead of eroding the masks by 2 pixels on BOLD space, the aCompCor masks are subtracted from a mask of pixels that likely contain a volume fraction of GM. This mask is obtained by thresholding the corresponding partial volume map at 0.05, and it ensures components are not extracted from voxels containing a minimal fraction of GM. Finally, these masks are resampled into BOLD space and binarized by thresholding at 0.99 (as in the original implementation). Components were also calculated separately within the WM and CSF masks. For each CompCor decomposition, the *k* components with the largest singular values were retained, such that the retained components' time series are sufficient to explain 50 percent of variance across the nuisance mask (CSF, WM, combined, or temporal). The remaining components are dropped from consideration. The head-motion estimates calculated in the correction step were also placed within the corresponding confounds file. The confound time series derived from head motion estimates and global signals were expanded with the inclusion of temporal derivatives and quadratic terms for each (S19). Frames that exceeded a threshold of 1.1 mm FD or 2.0 standardised DVARS were annotated as motion outliers. All resamplings were performed with *a single interpolation step* by composing all the pertinent transformations (i.e. head-motion transform matrices, susceptibility distortion correction when available, and co-registrations to anatomical and output spaces). Gridded (volumetric) resamplings were performed using antsApplyTransforms (ANTs), configured with Lanczos interpolation to minimize the smoothing effects of other kernels (S20). Non-gridded (surface) resamplings were performed using mri_vol2surf (FreeSurfer).

After preprocessing with fMRIprep, functional data were spatially smoothed with a Gaussian kernel of 6 x 6 x 6 mm full width at half maximum (FWHM) using the smoothing functionality in Statistical Parametric Mapping (SPM) 12 software version 7771 (The Wellcome Centre for Human Neuroimaging, University College London, UK) implemented in Matlab R2019b (Mathworks, Natick, MA, USA).

Statistical analysis

Behavioral data analysis

To explore potential moderators of the effect of emotion condition on physical symptom reporting, and to compare their moderating effects between groups, questionnaire scores were entered as continuous independent variables to the GEE models on physical symptom ratings, including all main effects and

interactions. The 3-way group x emotion condition x covariate effect was clarified by running the model in both groups separately.

fMRI data analysis

We used the neuroimaging analysis tools developed by the Cognitive and Affective Neuroscience Lab (CANlab) at Dartmouth College (https://github.com/canlab). These tools are written in Matlab (R2019b, Mathworks, Natick, MA, USA) and call Statistical Parametric Mapping (SPM12, v7771, Functional Imaging Laboratory (FIL), the Wellcome Centre for Human NeuroImaging (WCHN), Institute of Neurology, University College London (UCL), UK) functions.

First level analysis

fMRI data were analyzed at the subject level based on the general linear model (GLM) using Matlab script calling CANlab tools (https://github.com/labgas/proj-emosymp/tree/main/firstlevel). The first level design matrix *(Supplementary Fig. S1)* included one regressor for each level of affective valence (negative, neutral, and positive), one regressor for rating epochs per run, and the following nuisance regressors: (1) one regressor for the average CSF signal representing physiological noise, (2) 24 head movement parameter regressors (six parameters for translation and rotation in three directions, their squared values and the first-order derivatives for the raw and squared values), and (3) one regressor per spike volume identified based on the thresholds described above. Run intercepts were included, and a high pass filter of 180 sec was used.

Second level analysis

CANlab 2018 combined atlas:

The atlas defines a set of elementary brain areas by combining the Human Connectome Project's cortical parcellation with individual cerebellar lobules (S21, S22), and thalamic [S23-S25), amygdala and hippocampus (S26), basal ganglia (S27), and brainstem nuclear (S28-S36) parcellations. For details and Matlab code for this analysis method, see https://github.com/canlab/CanlabCore/blob/master/CanlabCore/%40fmri_data/robfit_parcelwise.m

Mediation analyses

Two complementary mediation analyses were performed using the CANlab mediation toolbox (https://github.com/canlab/MediationToolbox) (42). More specifically, the brain response was used as a mediator in the following two complementary ways. For both analyses, inference was performed using a bootstrap procedure with 5000 iterations. For details and Matlab code for these analyses, see https://github.com/labgas/proj-emosymp/blob/main/secondlevel/model_1_CANlab_classic_GLM/emosymp_m1_s6_mediation_NPS.m

(1) univariate mediation analysis with the NPS response as the mediator, i.e., Group → NPS → physical symptoms.
(2) whole-brain multivariate mediation analysis using the "principal directions of mediation" (PDM) method (43,44). Briefly, the PDM approach decomposes activity across the brain into multiple orthogonal networks that mediate group effects on physical symptom ratings. Contrary to (Chen et al. and Geuter et al.) (43,44), we used singular value decomposition (SVD) rather than population value decomposition (PVD) as an initial dimension reduction technique to accommodate the single- rather than multi-level nature of our analysis (i.e., between-subject analysis rather than single-trial within-subject analysis). As part of this procedure we chose to retain the first B=20 dimensions of the data prior to estimating the PDMs and path coefficients. Studying the absolute value of the indirect effect for each PDM shows a dramatic decrease between the second and third PDM. As the first two PDMs explain more than 90% of the total indirect effect we restrict further analysis to PDMs 1 and 2. PDM maps were thresholded at $q_{FDR}<0.05$. To render our models interpretable with respect to the neural processes under study, we apply source reconstruction, a method often applied to multivariate pattern analyses (S37), to our PDM data, as like MVPA models, path *b* represents a "backward model" in the terms of Haufe et al (S37). Briefly, we calculated the covariance between the observed activity in each voxel and estimates of latent factors computed as the product of mediation weights and brain activity for each of the two PDMs (as per equations 5 and 7 in Haufe et al.) (S37). The interpretation is then that voxels which show high covariance with mediation weights are voxels that encode the model's measure.

RESULTS

Participant characteristics

One control only completed five runs, and one run was excluded for a second control because this participant fell asleep during that run.

For six participants (two controls, four patients), one or more runs had to be excluded because of corrupt PAR/REC source data files, resulting in exclusion of 12 runs in total.

Participants in whom 4 or more of the runs were excluded based on the predefined quality control criteria (see supplement to Materials and Methods) were entirely excluded from the analysis, resulting in the exclusion of 3 patients. For the remaining participants, 47 runs were excluded in total (15 runs in controls, 32 in patients).

Marginal linear mixed model analysis on the included subjects with "group" as between-subject factor and "run" as within-subject factor, and including their interaction, did not reveal a significant difference

between controls and patients for standardized DVARS nor for mean FD (main effect of group $F_{1,58}$ = 0.71, p = 0.40, and $F_{1,58}$ = 3.09, p = 0.084, respectively).

Behavioral results

Physical symptoms

Moderation analyses – physical symptoms

Averages and SD of questionnaire scores in FSS patients and controls can be found in Supplementary table S1.

Habitual symptom reporting

A GEE model with negative binomial distribution was used to investigate the effect of habitual symptom reporting on state physical symptom ratings. Since controls were preselected to be low habitual symptom reporters, only the results of the moderation analysis in patients is reported here. On average, patients had a CDS score of 103.37 (SD = 23.93). A Box-Cox transformation was performed on CSD scores (λ = -0.75) prior to the GEE analysis. There was no significant main effect of habitual symptom reporting on physical symptom ratings [$\chi^2(1)$ = 0.16, p = 0.69], nor a significant habitual symptom reporting x emotion condition interaction effect [$\chi^2(2)$ = 1.28, p = 0.53].

Childhood trauma

A Box-Cox transformation was performed on the CTQ scores prior to analyses (λ = -1.5). A one-way ANOVA indicated no significant differences in CTQ scores in patients (observed mean = 41.83, SD = 17.26) versus controls (observed mean = 35.07, SD = 10.84; $F_{1,175}$ = 3.70, p = 0.056). A GEE model with negative binomial distribution was used to investigate the effect of childhood trauma experience on state physical symptom ratings. The main effect of CTQ scores on symptom ratings was non-significant [$\chi^2(1)$ = 0.12, p = 0.73], but there was a significant CTQ x emotion condition interaction effect [$\chi^2(2)$ = 6.56, p = 0.038] and a trend for a CTQ x emotion condition x group three-way interaction effect [$\chi^2(2)$ = 5.01, p = 0.082]. The CTQ x group interaction effect was not significant [$\chi^2(2)$ = 0.61, p = 0.43]. To facilitate the interpretation of the three-way interaction, the analysis was repeated on the groups separately. In the patient group, there was a significant CTQ x emotion condition interaction effect [$\chi^2(2)$ = 6.21, p = 0.045]. The main effect of CTQ scores on symptom ratings was not significant [$\chi^2(2)$ = 0.45, p = 0.51].

The planned comparisons indicated that for patients with low levels of childhood trauma there was no difference between symptom ratings in the different conditions (estimate LSM for patients with CTQ scores 1 SD below the average in the negative (2.58±0.074), neutral (2.68±0.061), and positive (2.73±0.086) emotion condition). Patients with average levels of childhood trauma had higher symptom ratings in the negative (LSM estimate 2.82±0.061) compared to the neutral (LSM estimate 2.63±0.037, p_{Holm} = 0.0004) and the positive (LSM estimate 2.66±0.051, p_{Holm} = 0.036) condition, while the latter

two did not differ ($p_{Holm} = 0.73$). Patients with high levels of childhood trauma (CTQ scores 1 SD above the average) had higher symptom ratings in the negative (LSM estimate 2.87±0.088) compared to the neutral (LSM estimate 2.58±0.030, $p_{Holm} = 0.0011$) and the positive (LSM estimate 2.57±0.050, $p_{Holm} = 0.0011$) condition, while the latter two did not differ ($p_{Holm} = 0.57$). In the controls group, both the main effect of CTQ scores [$\chi^2(2) = 0.16$, $p = 0.69$] and the CTQ x emotion interaction effect [$\chi^2(2) = 0.42$, $p = 0.81$] on symptom ratings were non-significant.

Difficulty Identifying Feelings

A Box-Cox transformation was performed on the score on the "difficulty identifying feelings" (DIF) subscale of the TAS-20 prior to analyses ($\lambda = 0$). Patients had a lower DIF score (observed mean = 13.23, SD = 3.65) than healthy controls (observed mean = 16.45, SD = 4.20, $F_{1,175} = 33.34$, $p < 0.0001$). A GEE model with negative binomial distribution was used to investigate the effect of DIF on state physical symptom ratings. The main effect of DIF [$\chi^2(1) = 0.44$, $p = 0.51$], the DIF x emotion condition interaction effect [$\chi^2(2) = 2.04$, $p = 0.36$], the DIF x group interaction effect [$\chi^2(1) = 2.50$, $p = 0.11$], and the DIF x emotion condition x group three-way interaction effect [$\chi^2(2) = 2.24$, $p = 0.32$] on symptom ratings were all non-significant.

Absorption

A Box-Cox transformation was performed on the Tellegen Absorption Scale scores prior to analyses ($\lambda = 0.75$). There was no significant difference on absorption scores in patients (observed mean = 12.03, SD = 6.76) versus healthy controls (observed mean = 13.10, SD = 7.34, $F_{1,175} = 0.98$, $p = 0.32$). A GEE model with negative binomial distribution was used to investigate the effect of absorption on state physical symptom ratings. The main effect of absorption [$\chi^2(1) = 0.37$, $p = 0.54$], the absorption x emotion condition interaction effect [$\chi^2(2) = 1.02$, $p = 0.60$], the absorption x group interaction effect [$\chi^2(1) = 0.07$, $p = 0.79$], and the absorption x emotion condition x group three-way interaction effect [$\chi^2(2) = 1.36$, $p = 0.51$] on symptom ratings were all non-significant.

fMRI results

Signature responses

Results for the negative > positive contrast are reported here.

Neurologic Pain Signature (NPS)

Entire pattern.

Similar trends to the negative > neutral contrast were found for the negative > positive contrast, in that the negative NPS response in controls was present but not significant (dot product -2.18 ± 1.34, $t(29) = -1.63$, $p = 0.11$, $d = -0.30$), with an absent response in patients (dot product 0.65 ± 0.86, $t(29) = 0.76$, p

= 0.45, d = 0.14), resulting in a between-group difference at trend level [t(49.5) = 1.78, p = 0.081) *(Supplementary Fig. S3a)*.

Subpatterns and subregions.

The NPS consists of regions with positive and negative predictive weights, hence we first split the pattern in two subpatterns accordingly. Results show that the abovementioned results for the NPS as a whole are driven by the subpattern with positive weights. More specifically, for the negative > positive contrast, the between-group difference was significant [t(56.8) = 2.37, p = 0.021], due to a positive response in patients (dot product 1.82 ± 1.02, t(29) = 1.79, p = 0.08, d = 0.33) and a negative response in controls (dot product -1.86 ± 1.18, t = -1.58, p = 0.12, d = -0.29) in the positive NPS subpattern *(Supplementary Fig. S4a).*

No significant between- nor within-group effects were found for the negative NPS subpattern. However, numerically stronger responses were found in controls compared to FSS patients (p = 0.11; *Supplementary Fig. S4b)*.

Picture-Induced Negative Emotion Signature (PINES)

For the negative > positive contrast, similar results to the negative > neutral contrast were found in that significant, albeit moderately sized, PINES activation was found in both patients (dot product 0.17 ± 0.07, t(29) = 2.26, p = 0.031, d = 0.41) and controls (dot product 0.18 ± 0.07, t(29) = 2.61, p = 0.014, d = 0.48), without a significant between-group difference [t(57.6) = -0.10, p = 0.92] *(Supplementary Fig. S3b)*.

Stimulus-Intensity Independent Pain Signature (SIIPS)

As for the negative > neutral contrast, no significant within group (patients: dot product 86.24 ± 108.69, t(29) = 0.79, p = 0.43, d = 0.15; controls: dot product 44.9±104.73, t(29) = 0.43, p = 0.67, d = 0.08), nor between-group [t(57.9 = 0.27, p = 0.79] effects were found for the negative > positive contrast, *(Supplementary Fig. S3c)*.

Mediation analyses

Principal directions of mediation (PDM)

We identified two independent brain activation patterns mediating the relationship between patient status (patient vs. HC) and negative-affect induced symptom reporting using the multivariate PDM analysis method.

For each of the two PDMs, the following paths were estimated as in a standard mediation model, and as in Geuter et al (44). Path *a* represents the effect of Group (X) on the brain mediator pattern response (M), with a positive sign indicating a higher brain response in patients in voxels with positive PDM weights (warm colors in brain figures) and less activity in voxels with negative PDM weights (cool colors in brain figures). Path *b* represents the association between the brain mediator pattern response (M) and the physical symptom response to the negative > neutral contrast (Y). A positive path *b* implies that voxels with positive weights contribute positively to the differential physical symptom rating after controlling for group. The absolute coefficient values for the indirect *ab* path indicate how much of the effect of group on physical symptom ratings is mediated through the PDM brain pattern. It should be noted that the (absolute) values of the path coefficients depend on the nature and scaling of the independent variable (X, which is an effects coded binary group variable, -1 for controls, 1 for patients), mediator (M, brain response), and dependent variable (Y, Z-scored differential rating variable). Hence, the fact that path *a* coefficients are three orders of magnitude larger than path *b* coefficients is related to scaling differences rather than their differential relevance/strength. It should also be noted that mixing of signals from distinct neural populations within fMRI voxels is common in similar types of analysis such as ICA, and can manifest itself in different weight patterns across PDMs, as illustrated by patterns in visual cortices in our study (see below).
When considering both the sign of path *b* (path *a* is constrained by the model to be always positive), and the voxel weights, four different possible relationships for the mediating clusters can be identified: (1) positive to group (i.e. more activation in patients), positive to physical symptoms; (2) negative to group (i.e. less activation in patients), negative to physical symptoms; (3) positive to group, negative to physical symptoms; (4) negative to group, positive to physical symptoms. Hence, (1) and (2) can be conceptualized as mediator effects, while (3) and (4) represent suppressor effects.

PDM 1

PDM 1 has a positive path *a* (23.61) and a negative path *b* (-0.015) coefficients, thereby acting as a suppressor of the group effect on negative-affect induced physical symptoms.

More specifically, voxels with positive weights correspond to scenario (3) above in that they show stronger responses to negative versus neutral pictures in patients versus controls, but a negative relationship with negative affect-induced physical symptoms while controlling for group. Hence, higher brain response is related to lower physical symptom ratings. As shown by warm colors in *Supplementary Fig. S5a and S6a,* and in *Supplementary Table S4*, such regions include vmPFC (BA 10), vlPFC (BA

47), fronto-parietal regions, posterior/mid insula, as well as medulla (vagal nuclei) and peripheral and central visual cortices.

Voxels with negative weights, on the other hand, correspond to scenario (4) above in that they show lower responses to negative versus neutral images in patients versus controls, with this lower activation being associated with more negative affect-induced physical symptoms. Such regions include ventral pons and dorsal midbrain (PAG area), cerebellar subregions, right anterior insula, precuneus (BA7)/retrosplenial cortex, (mostly peripheral) left visual cortices, and right temporal pole (area TGd/v), as shown by cool colors in *Supplementary Fig. S5a and S6a,* and in *Supplementary Table S4.*

Source reconstruction identifies a widely distributed pattern of voxels representing the mediation signal (i.e. their activity covarying with their mediation weight), shown in *Supplementary Fig. S7a*.

PDM 2

PDM 2 has positive path *a* (21.56) and path *b* (0.0126) coefficients, thereby acting as a mediator of the negative affect-induced physical symptoms.

More specifically, voxels with positive weights follow scenario (1) above with the higher response in patients corresponding to higher physical symptom ratings – hence acting as positive mediators - while controlling for group. Regions following such pattern include aMCC, somatomotor areas including S1, S2/operculum, dorsal posterior insula, dorsal pons/parabrachial nucleus, widespread cerebellar areas, temporal pole, peri/ectorhinal cortex (BA35, 36), dlPFC (BA8, 9, 46), precuneus (BA7), habenula, and peripheral and central visual cortices, as shown by warm colors in *Supplementary Fig. S5b and S6b,* and in *Supplementary Table S5.*

Voxels with negative weights in PDM 2 correspond to scenario (2) above, acting as negative mediators of the group – physical symptom relationship, in which less activation in patients compared to controls mediates higher symptoms. Such regions include (ventral) medulla, cerebellum (vermis, Crus I), and central and peripheral visual cortices, as shown by cool colors in *Supplementary Fig. S5b and S6b,* and in *Supplementary Table S5.*

Like for PDM 1, source reconstruction identifies a widely distributed pattern of voxels representing the mediation signal, shown in *Supplementary Fig. S7b*.

DISCUSSION

Discussion of behavioral moderation results

Interestingly, the effect of picture valence on physical symptom reporting was greater in patients who had experienced childhood trauma. This finding would fit with reduced detail in somatosensory processing of negative stimulation in individuals with traumatic experiences: a “quick and dirty” categorization of threatening information, representing a “better safe than sorry” processing strategy

during threat, precludes extensive sensory-perceptual information processing of both exteroceptive and interoceptive information (34). However, our sample size was too small to investigate whether the moderating role of childhood trauma was also visible in the imaging data. Also, this behavioral finding needs replication in a study that is sufficiently powered to investigate individual difference variables.

Discussion of PDM results

In addition to the NPS, we also identified two independent brain activation patterns mediating the relationship between patient status (patient vs. HC) and negative-affect induced symptom reporting using the multivariate PDM analysis method.

The "mediator" pattern (PDM 2) is the most straightforward to interpret. Patients demonstrated stronger negative affect-induced activation of regions known to be involved in (noxious) somatic stimulus processing, such as the aMCC, somatomotor areas (S1, S2/operculum, posterior insula), dorsal pons/parabrachial nucleus, and widespread cerebellar areas, but also in cognitive/affective modulation (dlPFC, ectorhinal cortex) of the perception of such stimuli, and stronger activation in these regions was related to higher physical symptoms, while controlling for Group (positive mediator). Several of these nociceptive regions are also key components of the NPS and were identified in our parcelwise GLM analysis as well, thereby corroborating and extending these abovementioned results. The "suppressor" pattern (PDM 1) exerts a more complex function. Patients demonstrated stronger negative affect-induced activation of, among others, regions involved in affect and pain modulation (vmPFC, vlPFC, frontoparietal areas), and interoceptive signaling/processing (vagal nuclei in the medulla, posterior insula), where stronger activation was related to less physical symptom reporting. Similarly, less strong activation of the PAG area (known to be involved in descending pain modulation), cerebellar subregions, and right anterior insula (a higher order area where interoceptive and affective input is integrated to shape conscious experience) was found in patients versus controls, with less strong activation being related to higher negative-affect induced symptom reporting. It should be noted that some of the non-nociceptive areas included in PDM1 (and to a lesser extent PDM2) such as vmPFC, dlPFC, dmPFC, and hippocampal subregions are also included in the SIIPS. This paints a more nuanced picture in that extra-nociceptive pain modulatory areas may also play a mediating role in negative-affect induced physical symptom reporting, in addition to the nociceptive areas covered by the NPS, although to a lesser extent (as reflected by the negative result for the SIIPS as a whole).

The fact that some regions, like insular and brainstem regions, appear in both mediator and suppressor relationships may come as a surprise, but has been observed before in the context of stimulus – pain relationships (44), and could be interpreted as mixing of signals from distinct neuronal populations within those regions. Moreover, as apparent from the comparison between Supplementary Fig. S5a and b, and S6a and b, there is limited overlap at the subregional/nuclear level. This phenomenon is perhaps most apparent in the visual cortex. Indeed, interestingly, but somewhat unexpectedly, peripheral and

central visual cortices figure prominently in both patterns. The occipital spatial patterns are complex and somewhat hard to interpret at the subregional level, although meta-analyses have shown that lateral occipital cortex is most robustly engaged during manipulations of affect, an effect which is thought to be driven by reentrant feedback from regions including amygdala and medial prefrontal cortex. Speculatively, the presence of positive loadings in both vmPFC and early visual cortices in PDM 1 could be consistent with such a feedback account. In general, our findings show that differences in response to the same negative affective images between patients and controls emerge already in early visual cortex and are involved in differential susceptibility to negative affect-induced physical symptoms.

SUPPLEMENTARY REFERENCES

1 A. Spruyt, J. Clarysse, D. Vansteenwegen, F. Baeyens, D. Hermans, Affect 4.0. A free software package for implementing psychological and psychophysiological experiments. *Exp Psychol* **57**, 36-45 (2009).

2 C.D. Scher, M.B. Stein, G.J. Asmundson, D.R. McCreary, D.R. Forde DR, The childhood trauma questionnaire in a community sample: psychometric properties and normative data. *J Trauma Stress* **14**, 843-57 (2001).

3 R.M. Bagby, J.D. Parker, G.J. Taylor, The twenty-item Toronto Alexithymia Scale—I. Item selection and cross-validation of the factor structure. *J Psychosom Res* **38**, 23-32 (1994).

4 A. Tellegen, G. Atkinson, Openness to absorbing and self-altering experiences (“absorption”), a trait related to hypnotic susceptibility. *J Abnorm Psychol* **83**, 268–277 (1974).

5 O. Esteban, C.J. Markiewicz, R.W. Blair, C.A. Moodie, A.I. Isik, A. Erramuzpe A, et al, fMRIPrep: a robust preprocessing pipeline for functional MRI. *Nat Methods* **16:**111-6 (2019).

6 O. Esteban, R. Blair, C.J. Markiewicz, S.L. Berleant, C.A. Moodie, F. Ma, K.J. Gorgolewski. FMRIPrep 1.1. 7. *Software* 2018.

7 K. Gorgolewski, C.D. Burns, C. Madison, D. Clark, Y.O. Halchenko, M.L. Waskom, S.S, Ghosh: a flexible, lightweight and extensible neuroimaging data processing framework in python. *Front Neuroinform* **22,** 13 (2011).

8 A. Abraham, F. Pedregosa, M. Eickenberg, P. Gervais, A. Mueller, J. Kossaifi, A. Gramfort, et al, Machine learning for neuroimaging with scikit-learn. *Front Neuroinform* **21**, 8-14 (2014).

9 N.J. Tustison, B.B. Avants, P.A. Cook, Y. Zheng, A. Egan, P.A. Yushkevich, J.C. Gee, N4ITK: improved N3 bias correction. *IEEE Trans Med Imaging* **29**, 1310-20 (2010).

10 B.B. Avants, C.L. Epstein, M. Grossman, J.C. Gee, Symmetric diffeomorphic image registration with cross-correlation: evaluating automated labeling of elderly and neurodegenerative brain. *Medical Image Analysis* **12**, 26-41 (2008).

11 Y. Zhang, M. Brady, S. Smith, Segmentation of brain MR images through a hidden Markov random field model and the expectation-maximization algorithm *IEEE Trans Med Imaging* **20**, 45-57 (2001).

12 V.S. Fonov, A.C. Evans, R.C. McKinstry, C.R. Almli, D.L. Collins, Unbiased nonlinear average age-appropriate brain templates from birth to adulthood. *NeuroImage* **47**, S102 (2009).

13 M. Jenkinson, S. Smith, A global optimisation method for robust affine registration of brain images. *Medical Image Analysis* **5**, 143-56 (2001).

14 D.N. Greve, B. Fischl, Accurate and robust brain image alignment using boundary-based registration. *NeuroImage* **48**, 63-72 (2009).

15 M. Jenkinson, P. Bannister, M. Brady, S. Smith, Improved optimization for the robust and accurate linear registration and motion correction of brain images. *NeuroImage* **17:**825-41 (2002).

16 R.W. Cox, J.S. Hyd, Software tools for analysis and visualization of fMRI data. *NMR Biomed* **10,** 171-8 (1997).

17 J.D. Power, A. Mitra, T.O. Laumann, A.Z. Snyder, B.L. Schlaggar, S.E. Petersen, Methods to detect, characterize, and remove motion artifact in resting state fMRI. *NeuroImage* **84,** 320-41 (2014).

18 Y. Behzadi, K. Restom, J. Liau, T.T. Liu, A component based noise correction method (CompCor) for BOLD and perfusion based fMRI. *NeuroImage* **37**, 90-101 (2007).

19 T.D. Satterthwaite, M.A. Elliott, R.T. Gerraty, K. Ruparel, J. Loughead, M.E. Calkins, et al, An improved framework for confound regression and filtering for control of motion artifact in the preprocessing of resting-state functional connectivity data. *NeuroImage* **64,** 240-56 (2013).

20 C. Lanczos. Evaluation of noisy data. *J SIAM Numer Anal* **1**, 76-85 (1964).

21 M.F. Glasser, T.S. Coalson, E.C. Robinson, C.D. Hacker, J. Harwell, E. Yacoub, et al, A multi-modal parcellation of human cerebral cortex. *Nat* **536,** 171-8 (2016).

22 J. Diedrichsen, J.H. Balsters, J. Flavell, E. Cussans, N. Ramnani, A probabilistic MR atlas of the human cerebellum. *Neuroimage* **46**:39-46 (2009).

23 A. Morel, M. Magnin, D. Jeanmonod. Multiarchitectonic and stereotactic atlas of the human thalamus. *J Comp Neurol* **387**, 588-630 (1997).

24 A. Krauth, R. Blanc, A. Poveda, D. Jeanmonod, A. Morel, G. Székely, A mean three-dimensional atlas of the human thalamus: generation from multiple histological data. *Neuroimage* **49**, 2053-62 (2010).

25 A. Jakab, R. Blanc, E.L. Berényi, G. Székely, Generation of individualized thalamus target maps by using statistical shape models and thalamocortical tractography. *Am J Neuroradiol* **33**, 2110-6 (2012).

26 K. Amunts, O. Kedo, M. Kindler, P. Pieperhoff, H. Mohlberg, N.J. Shah, et al, Cytoarchitectonic mapping of the human amygdala, hippocampal region and entorhinal cortex: intersubject variability and probability maps. *Anatom and embryol* **210,** 343-52 (2005).

27 W.M. Pauli, R.C. O'Reilly, T. Yarkoni, T.D. Wager, Regional specialization within the human striatum for diverse psychological functions. *PNAS* **113,** 1907-12 (2016).

28 G.A. Calvert, R. Campbell, M.J. Brammer, Evidence from functional magnetic resonance imaging of crossmodal binding in the human heteromodal cortex. *Curr Biol* **10,** 649-57 (2000).

29 N.I. Keren, C.T. Lozar, K.C. Harris, P.S. Morgan, M.A. Eckert. In vivo mapping of the human locus coeruleus. *Neuroimage* **47**, 1261-7 (2009).

30 P.G. Nash, V.G. Macefield, I.J. Klineberg, G.M. Murray, L.A. Henderson, Differential activation of the human trigeminal nuclear complex by noxious and non-noxious orofacial stimulation. *Hum Brain Mapp* **30**, 3772-82 (2009).

31 K.J. Bär, F. de la Cruz, A. Schumann, S. Koehler, H. Sauer, H. Critchley, et al. Functional connectivity and network analysis of midbrain and brainstem nuclei. *Neuroimage* **134**:53-63 (2016).

32 V. Beliveau, C. Svarer, V.G. Frokjaer, G.M. Knudsen, D.N. Greve, P.M. Fisher, Functional connectivity of the dorsal and median raphe nuclei at rest. *Neuroimage* **116**, 187-95 (2015).

33 M. Fairhurst, K. Wiech, P. Dunckley, I. Tracey, Anticipatory brainstem activity predicts neural processing of pain in humans. *Pain* **128**, 101-10 (2007).

34 J.C. Brooks, W.E. Davies, A.E. Pickering. Resolving the brainstem contributions to attentional analgesia. *J Neurosci* **37**, 2279-91 (2017).

35 R. Sclocco, F. Beissner, G. Desbordes, J.R. Polimeni, L.L. Wald, N.W. Kettner, et al, Neuroimaging brainstem circuitry supporting cardiovagal response to pain: a combined heart rate variability/ultrahigh-field (7T) functional magnetic resonance imaging study. *Philos Trans A Math Phys Eng Sci* **374**, 20150189 (2016).

36 X. Shen, F. Tokoglu, X. Papademetris, R.T. Constable, Groupwise whole-brain parcellation from resting-state fMRI data for network node identification. *Neuroimage* **82**, 403-15 (2013).

37 Haufe S, Meinecke F, Görgen K, Dähne S, Haynes JD, Blankertz B, Biessmann F. On the interpretation of weight vectors of linear models in multivariate neuroimaging. *Neuroimage 2014;* **87**:96-110.

SUPPLEMENTARY FIGURES

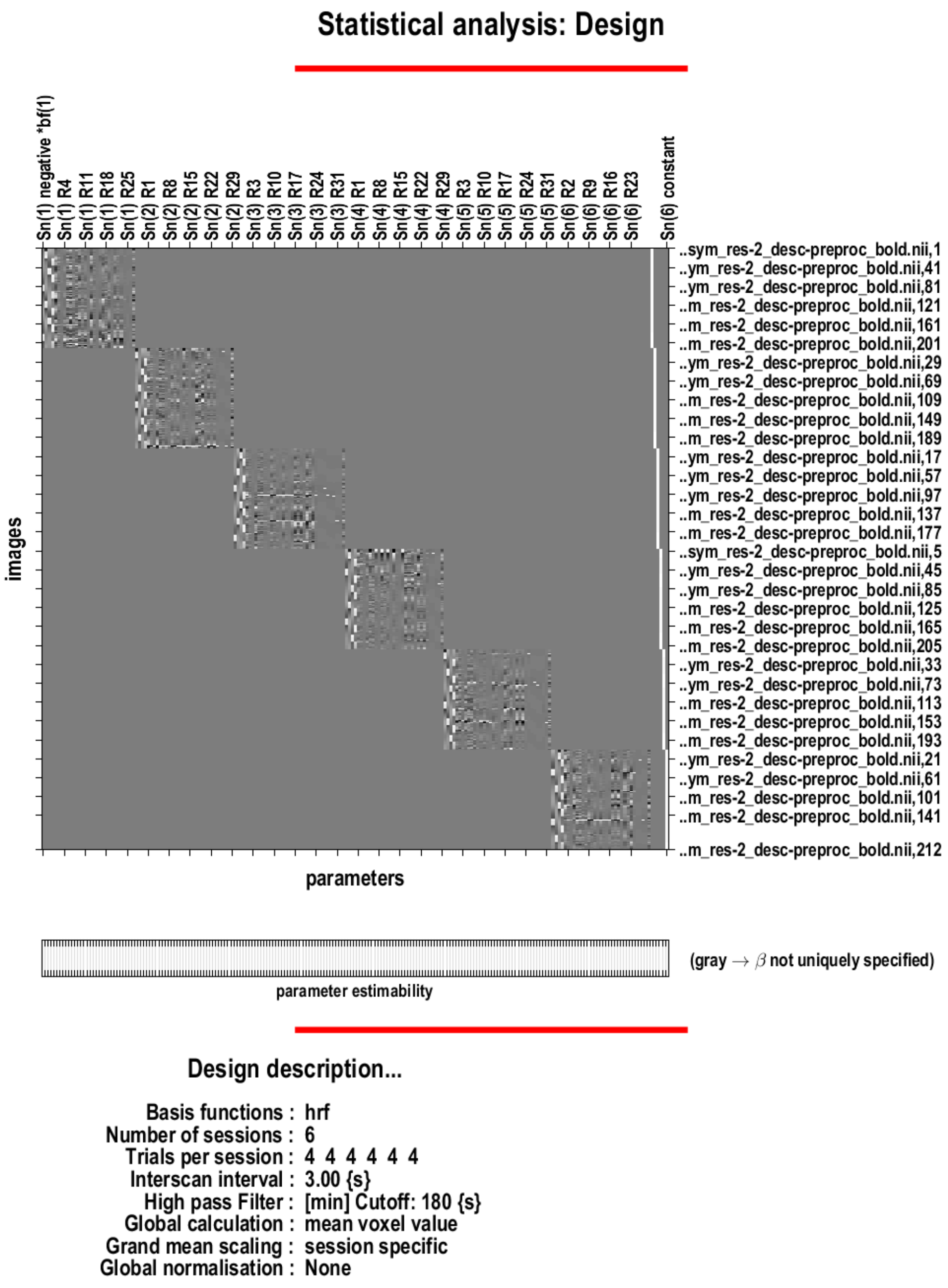

Supplementary Fig. S1. First level design matrix

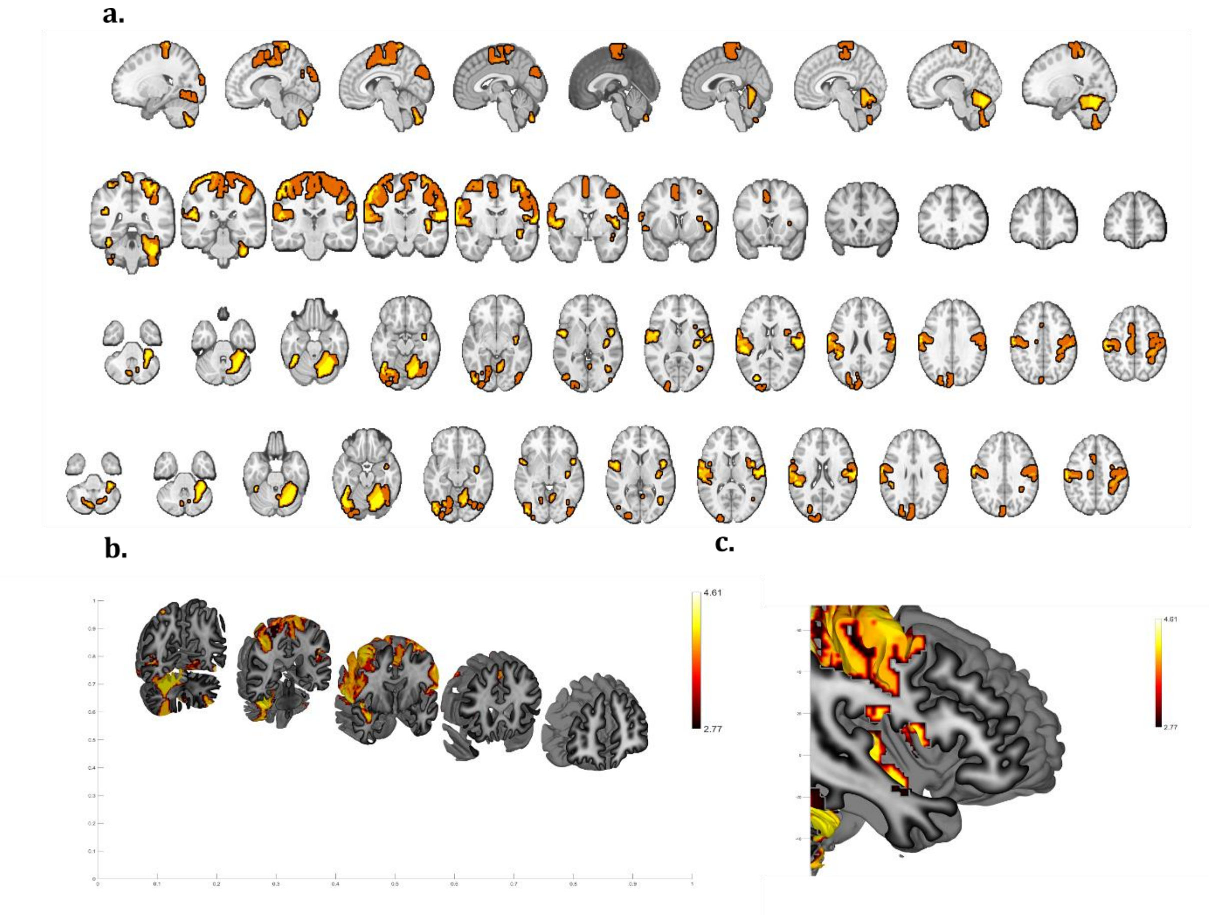

Supplementary Fig. S2. Supplement to whole brain parcelwise results, for the contrast negative > neutral in patients vs. HC. (a) Montage of sagittal, coronal and axial brain slices; (b,c) coronal and sagittal detail views of the somatomotor and insular clusters.

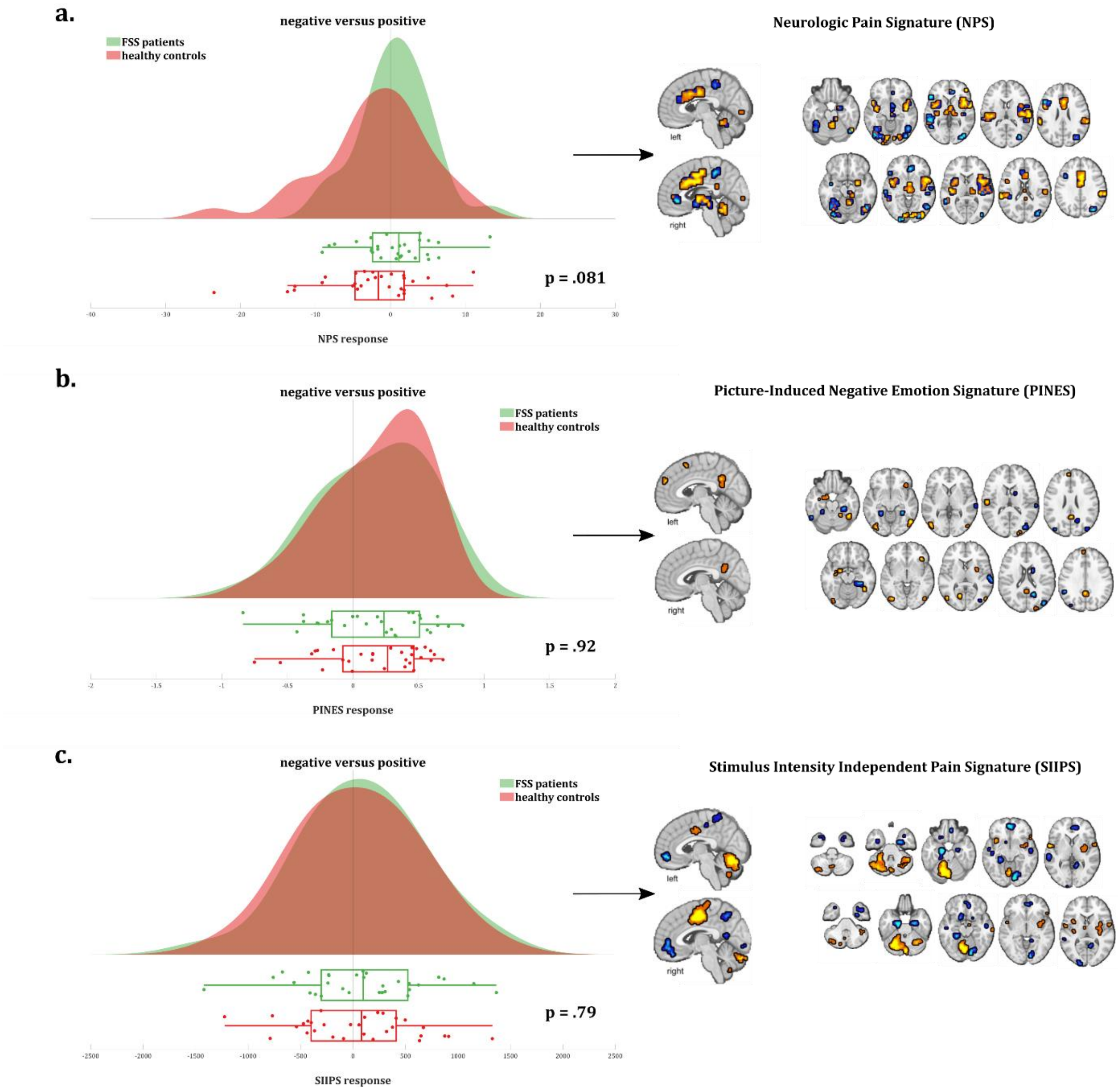

Supplementary Fig. S3. (a) NPS response, (b) PINES response, and (c) SIIPS response in functional somatic syndrome patients vs. healthy controls for the negative > positive valence contrast, including visual representation of the three neural signatures.

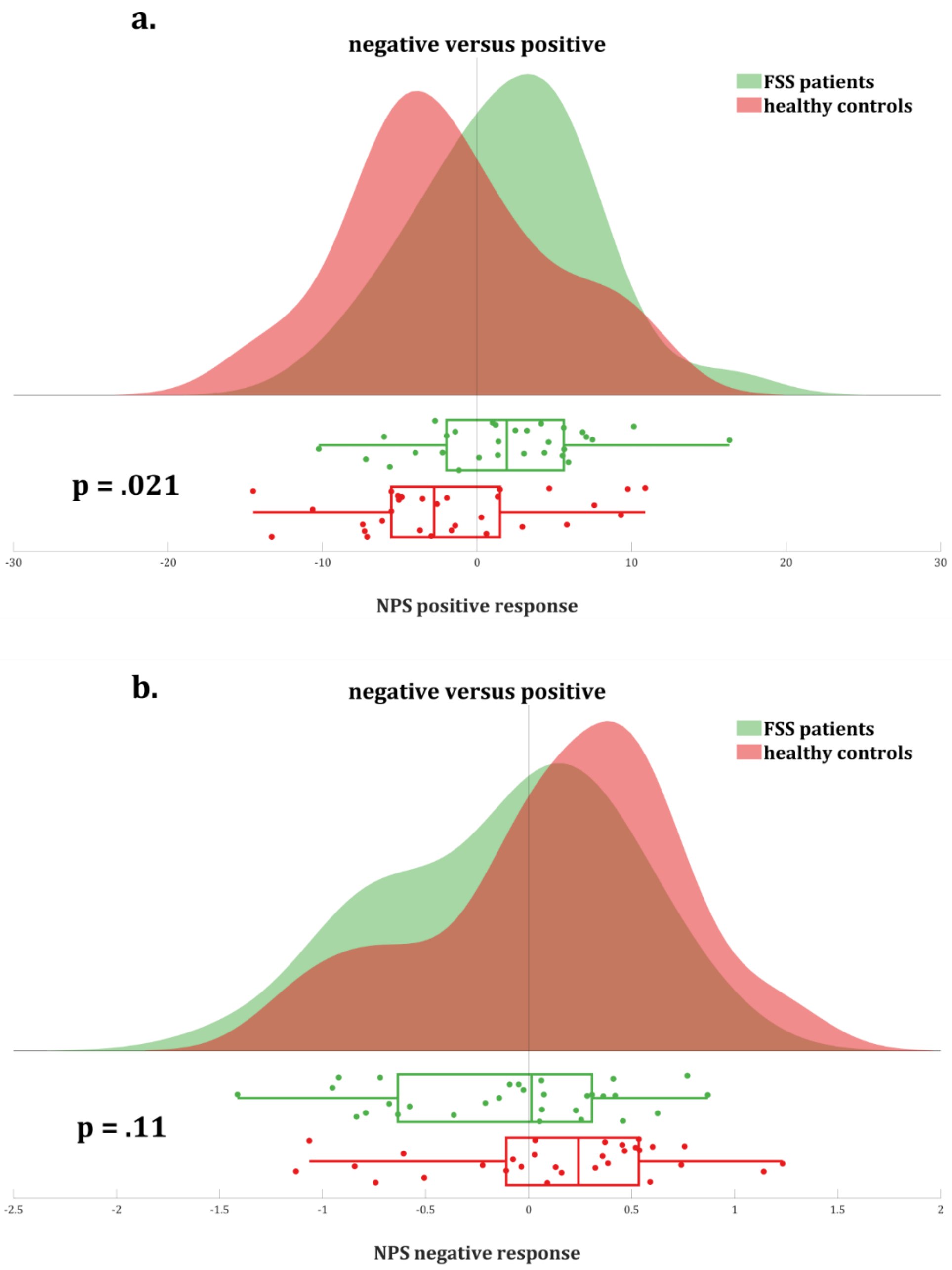

Supplementary Fig. S4. Brain response to negative > positive valence in the NPS subpatterns with (a) positive and (b) negative weights in functional somatic syndrome patients vs. healthy controls.

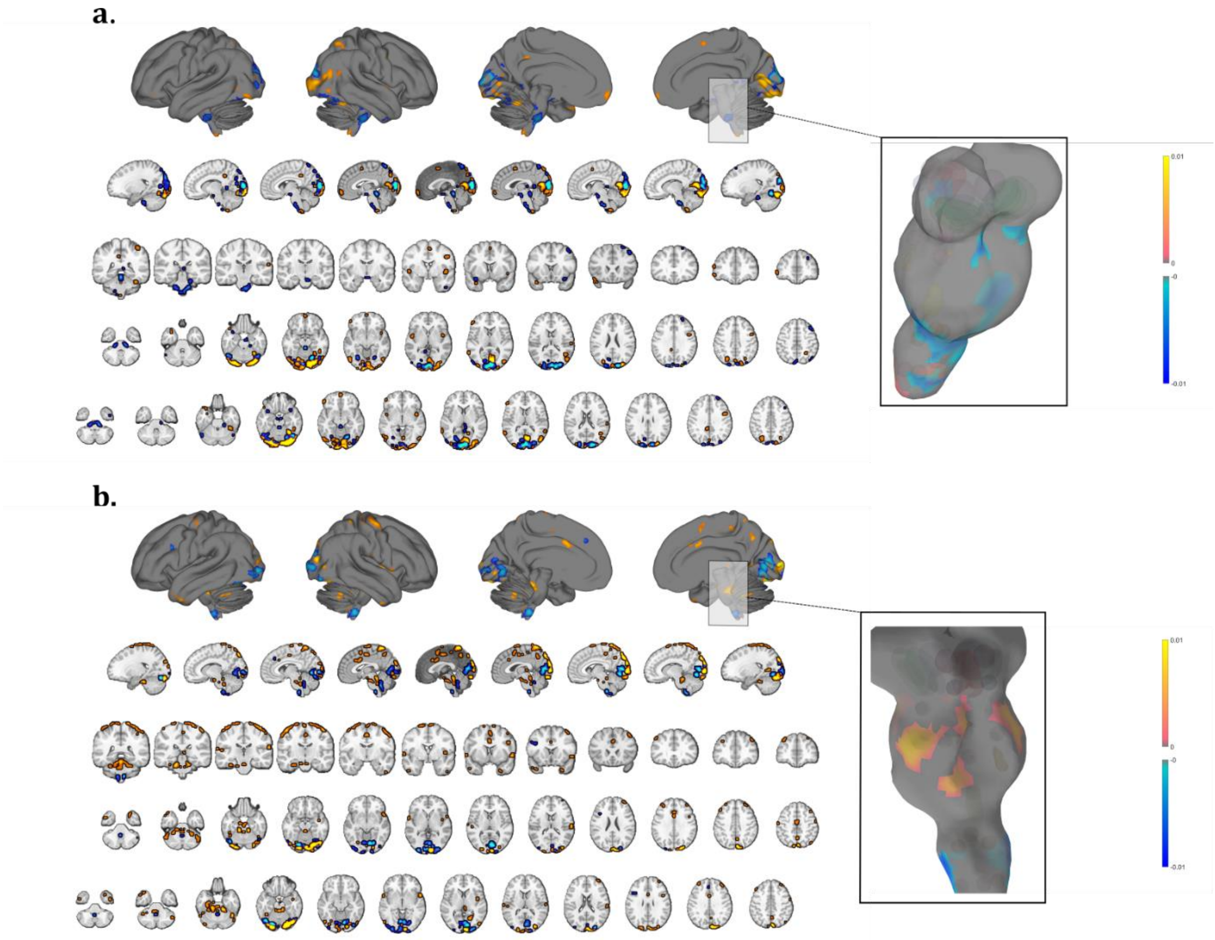

Supplementary Fig. S5. Independent brain activation patterns [(a) PDM 1 ("suppressor"); (b) PDM 2 ("mediator")] identified as mediators of the relationship between patient status [functional somatic syndrome (FSS) patient vs. healthy control] and physical symptom ratings after negative vs. neutral picture viewing.

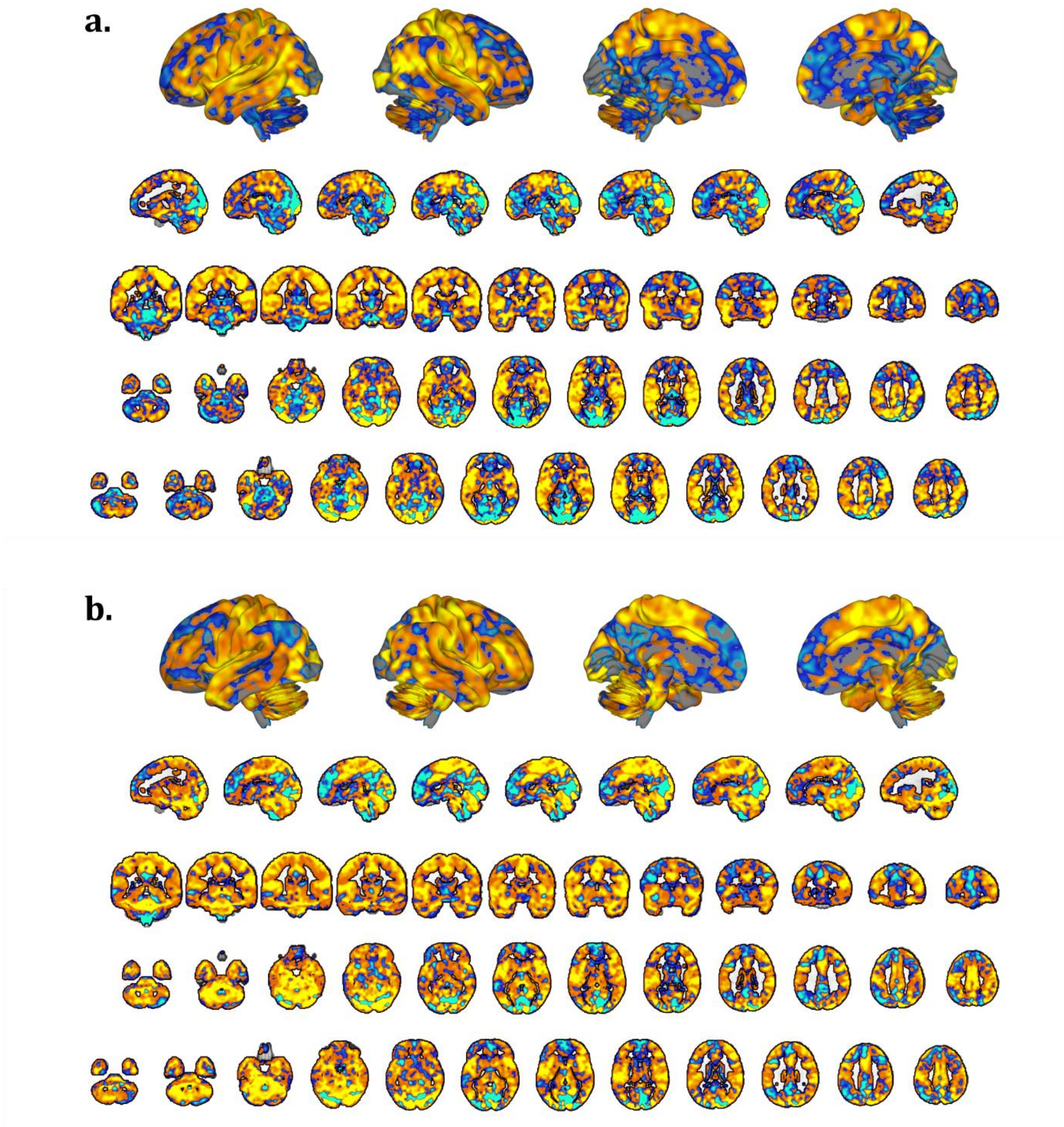

Supplementary Fig. S6. Supplement to independent brain activation patterns [(a) PDM 1 (“suppressor”); (b) PDM 2 (“mediator”)] identified as mediators of the relationship between patient status [functional somatic syndrome (FSS) patient vs. healthy control] and physical symptom ratings after negative vs. neutral picture viewing.

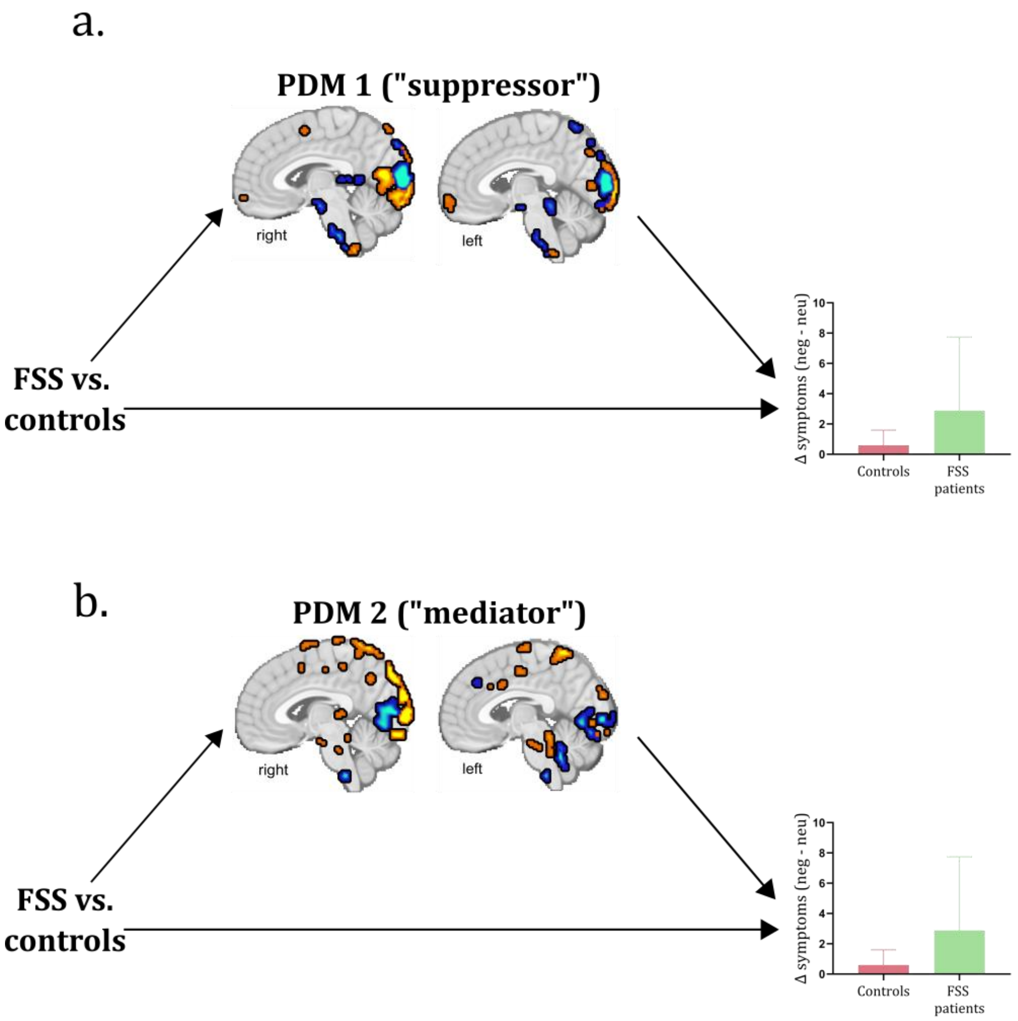

Supplementary Fig. S7. Supplement to independent brain activation patterns identified as mediators of the relationship between patient status [functional somatic syndrome (FSS) patient vs. healthy control] and physical symptom ratings after negative vs. neutral picture viewing. Unthresholded source reconstruction maps representing (a) PDM 1 ("suppressor") and (b) PDM 2 ("mediator).

Supplementary table S1. Comparison of trait questionnaire scores between FSS patients and controls. Trait questionnaire scores were boxcox-transformed before performing t-tests.

	**FSS patients**		**Controls**				
	**Mean**	**SD**	**Mean**	**SD**	**t**	**df**	**p**
Habitual symptom reporting (CSD)	103.37	23.93	60.65	7.35	11.54	58	<0.001
Childhood trauma (CTQ)	41.83	17.27	35.07	10.84	1.10	57	0.27
Difficulty identifying feelings (TAS20 – DIF)	13.07	3.58	16.34	4.18	3.36	57	0.001
Absorption (TAS)	12.66	6.19	11.63	7.43	0.68	57	0.50

Supplementary table S2. Significant (at qFDR < 0.05) regions from the CANlab 2018 combined atlas comparing patients vs. controls in the negative vs. neutral contrast.

Region	Volume	X	Y	Z	maxZ	Modal Label Descriptions	# Atlas Regions Covered	(Additional) Atlas Regions Covered
**PATIENTS > CONTROLS**								
Cblm_VIIb_L	9080	-26	-68	-54	3,291013956	Cerebellum	1	
Cblm_VI_R	35760	24	-56	-30	3,419451952	Cerebellum	5	Ctx_V8_R; Ctx_FFC_R; Cblm_V_R; Cblm_VIIIa_R
Multiple regions	14424	-32	-72	-6	4,307130337	Cortex_Visual_Central	6	Ctx_V3_L; Ctx_V8_L; Ctx_FFC_L; Ctx_LO2_L; Ctx_PIT_L; Ctx_V4t_L
Ctx_PoI1_R	1360	38	-14	-2	3,436470032	Cortex_Ventral_AttentionA	1	
Ctx_LO2_R	920	44	-82	-4	2,855539799	Cortex_Visual_Central	1	
Ctx_FOP1_R	1800	44	4	8	3,531669855	Cortex_Ventral_AttentionA	2	Ctx_FOP3_R
Ctx_MST_R	520	40	-64	4	3,297605515	Cortex_Dorsal_AttentionA	1	
Multiple regions	90240	-4	-20	48	3,944728851	Cortex_SomatomotorA	23	Ctx_4_L; Ctx_4_R; Ctx_3b_L; Ctx_3b_R; Ctx_FEF_R; Ctx_24dd_L; Ctx_SCEF_L; Ctx_7PC_R; Ctx_1_L; Ctx_1_R; Ctx_2_R; Ctx_3a_L; Ctx_6d_L; Ctx_6d_R; Ctx_43_L; Ctx_OP4_L; Ctx_OP4_R; Ctx_OP1_L; Ctx_PFcm_L; Ctx_FOP1_L; Ctx_AIP_R; Ctx_PFop_L; Ctx_PFop_R
Ctx_V7_L	1568	-28	-84	24	4,61361742	Cortex_Visual_Central	2	Ctx_V3B_L
**CONTROLS > PATIENTS**								
No significant parcels at qFDR < 0.05								

Regions from the CANlab 2018 combined atlas (https://sites.google.com/dartmouth.edu/canlab-brainpatterns/brain-atlases-and-parcellations/2018-combined-atlas), including the cortical atlas from (Glasser et al, Nature 2016) and the cerebellar atlas from (Diedrichsen et al, NeuroImage 2009). FFC, Fusiform Face Complex; LO2, lateral occipital area; PIT, posterior inferotemporal complex; PoI, posterior insular area; FOP, frontal opercular area; MST, medial superior temporal area: FEF, frontal eye fields; SCEF, supplementary and cingulate eye field; AIP, anterior intraparietal area; PFcm, area PFcm (inferior parietal lobule); PFop, area PF opercular (inferior parietal lobule)

Supplementary table S3. Summary of the NPS, PINES and SIIPS response during negative and neutral picture viewing in FSS patients and controls.

	FSS patients				Controls				Negative vs. neutral in FSS patients vs. controls		
	**Negative pictures**		**Neutral pictures**		**Negative pictures**		**Neutral pictures**				
	Mean	SE	Mean	SE	Mean	SE	Mean	SE	t	df	p
NPS response	-9.447	1.280	-12.224	1.172	-9.159	1.162	-9.948	1.159	2.30	51.3	0.026
PINES response	1.017	0.156	0.481	0.146	0.812	0.110	0.306	0.117	0.23	54.4	0.82
SIIPS response	-1963.4	142.01	-1942.6	149.06	-1874.0	144.65	-1899.0	146.19	0.03	57.9	0.97

Supplementary table S4. Regions from the CANlab 2018 combined atlas that constituting PDM 1 - "suppressor" pattern – mediating the group effect on negative-affect induced physical symptoms.

Region	Volume	X	Y	Z	maxZ	Modal Label Descriptions	# Atlas Regions Covered	(Additional) Atlas Regions Covered
**POSITIVE REGIONS**								
Cblm_VIIIb_R	1768	6	-47	-63	0,009321555728	Cerebellum	2	dmnx_nts_L; nuc_ambiguus_R
dmnx_nts_L	56	-5	-37	-65	0,004570126236	Brainstem	0	
dmnx_nts_L	64	-1	-37	-65	0,006883237086	Brainstem	0	
Cblm_VIIIb_L	880	-13	-49	-61	0,008925127208	Cerebellum	0	
Ctx_TGd_L	808	-35	16	-29	0,005992860596	Cortex_Default_ModeB	0	
Cblm_VI_R	816	40	-41	-27	0,008343746922	Cerebellum	0	
Ctx_TGd_L	456	-45	22	-23	0,004743791963	Cortex_Default_ModeB	0	
Multiple regions	39344	10	-85	-13	0,03760783923	Cerebellum	10	Ctx_PIT_L; Ctx_LO3_R; Ctx_LO1_R; Ctx_PIT_R; Ctx_V4_R; Ctx_V2_R; Ctx_V8_R; Ctx_LO2_L; Ctx_TPOJ3_R; Ctx_LO2_R
Cblm_VI_R	1920	32	-53	-19	0,01466060197	Cerebellum	1	Ctx_VVC_R
Ctx_TE2p_R	544	46	-47	-17	0,005456431973	Cortex_Dorsal_AttentionA	0	
Ctx_10v_L	1192	-1	64	-11	0,006481783058	Cortex_Limbic	0	
Ctx_a47r_L	896	-51	44	-9	0,00585629099	Cortex_Fronto_ParietalB	0	
Ctx_V2_L	288	-29	-101	-1	0,007160931642	Cortex_Visual_Peripheral	0	
Ctx_V1_R	6024	8	-75	10	0,02104480923	Cortex_Visual_Peripheral	1	
Ctx_PoI2_R	784	46	2	-3	0,006221065844	Cortex_Ventral_AttentionA	0	
Ctx_TA2_L	656	-49	-1	2	0,005262725592	Cortex_Temporal_Parietal	0	
Ctx_V1_R	392	14	-57	8	0,006074392707	Cortex_Visual_Peripheral	0	
Ctx_STV_L	1128	-65	-53	10	0,0058444672	Cortex_Temporal_Parietal	0	
Ctx_V1_R	56	8	-103	6	0,006964036104	Cortex_Visual_Peripheral	0	
Ctx_V3CD_L	1248	-35	-91	12	0,006811949534	Cortex_Visual_Central	1	
Ctx_IFSa_L	656	-53	40	12	0,005806375261	Cortex_Fronto_ParietalA	0	

Ctx_TPOJ2_R	416	62	-51	14	0,00486899121	Cortex_Dorsal_AttentionA	0	
Ctx_PGi_R	896	46	-57	18	0,006419657588	Cortex_Default_ModeC	0	
Ctx_OP4_R	520	64	-25	20	0,004759837704	Cortex_SomatomotorB	0	
Ctx_V2_R	56	12	-101	20	0,004575883383	Cortex_Visual_Central	0	
Ctx_V3_L	768	-1	-93	26	0,007515922455	Cortex_Visual_Central	0	
Ctx_V2_R	104	10	-99	24	0,005940304014	Cortex_Visual_Central	0	
Ctx_IP0_L	1392	30	-89	28	0,01812179321	Cortex_Visual_Peripheral	1	
Ctx_V3_R	552	-29	-81	28	0,006177771171	Cortex_Dorsal_AttentionA	0	
Ctx_31pv_L	168	4	-93	32	0,006319727756	Cortex_Visual_Central	0	
Ctx_PEF_R	448	-7	-41	36	0,00521958212	Cortex_Default_ModeA	0	
Ctx_POS2_L	848	50	4	36	0,006516259131	Cortex_Fronto_ParietalA	1	
Ctx_V6_R	456	-1	-77	40	0,006335202544	Cortex_Fronto_ParietalC	0	
Ctx_IP1_L	936	18	-85	42	0,006759542004	Cortex_Visual_Peripheral	0	
Ctx_IP1_R	1408	-27	-73	42	0,00679252417	Cortex_Fronto_ParietalA	2	Ctx_IPS1_L
Ctx_LIPv_R	408	30	-71	42	0,005236708401	Cortex_Dorsal_AttentionA	0	
Ctx_7Pm_R	1608	26	-59	54	0,007322232942	Cortex_Dorsal_AttentionA	1	
Ctx_SCEF_R	480	4	-75	56	0,007266667109	Cortex_Fronto_ParietalC	0	
Ctx_2_R	616	4	6	56	0,005323224196	Cortex_Ventral_AttentionA	0	
Ctx_VIP_L	720	44	-39	60	0,005912368807	Cortex_Dorsal_AttentionB	0	
	616	-23	-67	62	0,005386931791	Cortex_Dorsal_AttentionA	0	
**NEGATIVE REGIONS**								
Bstem_Ponscv_R	6936	2	-27	-49	-0,01549668888	Brainstem	5	nuc_ambiguus_R; Cblm_X_L; Bstem_Med_R; Bstem_Med_L
Ctx_TGv_R	320	46	-1	-41	-0,004924243559	Cortex_Limbic	0	
Cblm_CrusI_L	3584	-43	-65	-21	-0,01102717061	Cerebellum	1	
Cblm_I_IV_R	608	20	-31	-27	-0,007334692307	Cerebellum	0	
Cblm_VI_R	6304	28	-67	-17	-0,01991305129	Cerebellum	1	
Bstem_Midb_R	1248	4	-11	-19	-0,0104068453	Brainstem	1	
Ctx_TGd_L	448	-35	12	-21	-0,006613841331	Cortex_Default_ModeB	0	
Ctx_V1_L	26400	2	-93	12	-0,02464164671	Cortex_Visual_Peripheral	4	Ctx_V3A_L; Ctx_V3A_R; Ctx_V7_L; Ctx_V6A_L
Cblm_I_IV_L	1272	-1	-39	-15	-0,01171501728	Cerebellum	2	IC_L; Median_raphe_MR_R
Ctx_AAIC_R	352	36	16	-19	-0,004960132467	Cortex_Ventral_AttentionA	0	

Ctx_V1_L	80	-3	-97	-9	-0,006571580007	Cortex_Visual_Peripheral	0	
Ctx_V4_L	1024	-29	-69	-7	-0,007706727044	Cortex_Visual_Central	0	
Ctx_RSC_L	424	-11	-49	2	-0,005578287161	Cortex_Default_ModeC	0	
Ctx_LO3_L	784	-51	-83	8	-0,008523472388	Cortex_Visual_Central	1	
Ctx_V1_L	2392	-17	-77	10	-0,007623925612	Cortex_Visual_Peripheral	0	
Cblm_I_IV_R	408	4	-49	6	-0,005822512172	Cerebellum	0	
Ctx_V6_L	576	-11	-75	28	-0,005518214783	Cortex_Visual_Peripheral	0	
Ctx_9p_R	448	26	52	34	-0,004739240974	Cortex_Default_ModeB	0	
Ctx_V3_L	288	-5	-89	36	-0,005996778778	Cortex_Visual_Central	0	
Ctx_PGp_R	456	34	-83	36	-0,006307752031	Cortex_Default_ModeC	0	
Ctx_7PL_L	1552	-11	-83	50	-0,008682854391	Cortex_Dorsal_AttentionA	0	
Ctx_8Av_R	1120	44	22	50	-0,006025822226	Cortex_Fronto_ParietalB	0	
Ctx_PGs_R	848	42	-71	52	-0,006037213343	Cortex_Default_ModeA	0	
Ctx_POS2_R	288	14	-81	52	-0,005876144476	Cortex_Fronto_ParietalC	0	
Ctx_s6_8_R	864	28	24	60	-0,00556011303	Cortex_Default_ModeA	0	
Ctx_7Am_L	1464	-1	-61	64	-0,009374863495	Cortex_Dorsal_AttentionB	0	

Regions from the CANlab 2018 combined atlas (https://sites.google.com/dartmouth.edu/canlab-brainpatterns/brain-atlases-and-parcellations/2018-combined-atlas), including the cortical atlas from (Glasser et al, Nature 2016), brainstem areas from (Shen et al, 2013), and the cerebellar atlas from (Diedrichsen et al, NeuroImage 2009). dmnx_nts, dorsal motor nucleus of the vagus, nucleus of the solitary tract; nuc_ambiguus, nucleus ambiguus; TGd, area TG dorsal; PIT, posterior inferotemporal complex; LO, lateral occipital; TPOJ, temporoparietal junction; VVC, ventral visual complex; TE2p, area TE2 posterior; PoI2, posterior insular area 2; TA2, area TA2; STV, superior temporal visual area; V3CD, area V3CD; IFSa, area IFSa; TPOJ2n, TemporoParietoOccipital Junction 2; PG, area PG; IP, intraparietal area; PEF, premotor eye field; POS, parieto-occipital sulcus; LIP, lateral intraparietal; SCEF, supplementary & cingulate eye field; VIP, ventral intraparietal complex; TG, area TG; AAIC, anterior agranular insula complex; RSC, retrosplenial cortex; IC, inferior colliculus.

Supplementary table S5. Regions from the CANlab 2018 combined atlas that constituting DM 2 - "mediator" pattern – mediating the group effect on negative-affect induced physical symptoms.

Region	Volume	X	Y	Z	maxZ	Modal Label Descriptions	# Atlas Regions Covered	(Additional) Atlas Regions Covered
**POSITIVE REGIONS**								
Cblm_VIIIa_L	376	-31	-49	-49	0,004991472838	Cerebellum	1	
Cblm_VIIIb_R	496	28	-47	-47	0,005864710914	Cerebellum	1	
Ctx_TGv_R	560	48	-3	-43	0,006816107935	Cortex_Limbic	1	
Cblm_Dentate_L	312	-13	-49	-41	0,005268197858	Cerebellum	1	
Ctx_TGd_L	1240	-47	8	-37	0,006719851128	Cortex_Default_ModeB	1	
Ctx_TGd_R	792	28	14	-41	0,005550781842	Cortex_Limbic	1	
Cblm_CrusI_R	2304	48	-57	-29	0,008282561769	Cerebellum	1	
Cblm_I_IV_R	5192	14	-37	-25	0,00947974985	Cerebellum	4	pbn_R; lc_L; Median_raphe_MR_R
Ctx_TGd_L	408	-37	18	-35	0,006313357609	Cortex_Default_ModeB	1	
Cblm_CrusI_L	1920	-47	-53	-31	0,00641559168	Cerebellum	1	
Cblm_VI_L	1056	-27	-41	-29	0,005424653891	Cerebellum	1	
Cblm_I_IV_L	4128	-11	-31	-25	0,008763238686	Cerebellum	2	Bstem_Ponsrd_L
Bstem_Ponscd	552	-11	-13	-23	0,006705867305	Brainstem	1	
Cblm_CrusI_L	5112	-25	-87	-19	0,02513384886	Cerebellum	1	
Ctx_PeEc_L	1080	-31	-19	-25	0,006585102857	Cortex_Limbic	1	
Cblm_CrusI_R	10208	28	-81	-17	0,02799383957	Cerebellum	3	Ctx_PIT_R; Ctx_V8_R
Ctx_FFC_L	392	-41	-49	-21	0,006385918098	Cortex_Dorsal_AttentionA	1	
Ctx_PoI1_R	328	42	-3	-17	0,005562753841	Cortex_Ventral_AttentionA	1	
Ctx_PIT_L	576	-47	-75	-13	0,007446170502	Cortex_Visual_Central	1	
Ctx_V8_L	464	-27	-71	-9	0,00551352027	Cortex_Visual_Central	1	
Ctx_V2_L	296	-1	-83	-5	0,006851492133	Cortex_Visual_Peripheral	1	
Ctx_TA2_R	1240	56	12	-3	0,007581145231	Cortex_SomatomotorB	1	
Ctx_V1_R	10904	10	-93	24	0,02317948706	Cortex_Visual_Peripheral	3	Ctx_V3A_R; Ctx_V7_R
Ctx_FST_L	312	-55	-73	-3	0,005829875597	Cortex_Fronto_ParietalA	1	
Ctx_A4_L	736	-59	6	-1	0,005849548481	Cortex_SomatomotorB	1	

Ctx_PoI2_R	592	44	-1	6	0,006096953991	Cortex_Ventral_AttentionA	1	
Ctx_LO3_R	504	48	-79	6	0,006840260275	Cortex_Visual_Central	1	
Ctx_V1_R	1072	22	-61	6	0,006822027903	Cortex_Visual_Peripheral	1	
Haben	336	6	-31	6	0,005642451672	Diencephalon	1	
Ctx_V2_L	616	-7	-101	12	0,007274574193	Cortex_Visual_Peripheral	1	
Ctx_V3_L	1032	-23	-97	14	0,008222583371	Cortex_Visual_Central	1	
Ctx_V4_R	1096	32	-87	14	0,0106119445	Cortex_Visual_Central	1	
Ctx_PBelt_L	320	-55	-25	12	0,005268346828	Cortex_SomatomotorB	1	
Ctx_OP4_L	352	-67	-21	14	0,005652211251	Cortex_SomatomotorB	1	
Ctx_OP4_R	1368	64	-23	18	0,007479031993	Cortex_SomatomotorB	1	
Ctx_V2_R	32	18	-101	18	0,004908215407	Cortex_Visual_Central	1	
Ctx_9_46d_R	920	36	54	24	0,006196738675	Cortex_Ventral_AttentionB	1	
Ctx_V7_L	808	-21	-91	30	0,006964534013	Cortex_Visual_Central	1	
Ctx_6r_R	424	48	10	30	0,006072825631	Cortex_Ventral_AttentionA	1	
Ctx_a24pr_L	1280	-1	14	34	0,006249953625	Cortex_Ventral_AttentionB	2	Ctx_33pr_L
Ctx_46_L	824	-33	46	36	0,005828934178	Cortex_Ventral_AttentionB	1	
Ctx_p9_46v_R	1000	38	42	38	0,006648215109	Cortex_Fronto_ParietalA	1	
Ctx_7m_R	1080	4	-59	46	0,00616314775	Cortex_Default_ModeA	1	
Ctx_8C_L	392	-47	30	42	0,005599707521	Cortex_Fronto_ParietalA	1	
Ctx_7PL_L	1360	-9	-79	54	0,01036058331	Cortex_Dorsal_AttentionA	1	
Ctx_PGs_R	1080	44	-69	52	0,005985992991	Cortex_Default_ModeA	1	
Multiple regions	33832	6	-29	78	0,01087112543	Cortex_SomatomotorA	12	Ctx_5L_R; Ctx_5L_L; Ctx_6d_R; Ctx_6d_L; Ctx_1_R; Ctx_1_L; Ctx_7Am_R; Ctx_7PC_L; Ctx_7PC_R; Ctx_FEF_R; Ctx_VIP_R
Ctx_8Av_R	400	42	24	48	0,005719407412	Cortex_Fronto_ParietalB	1	
Ctx_24dd_L	1176	-1	-9	50	0,00678109191	Cortex_SomatomotorA	1	
Ctx_5m_R	512	6	-35	54	0,005211307528	Cortex_SomatomotorA	1	
**NEGATIVE REGIONS**								
Ctx_SCEF_R	904	2	8	56	0,005835505167	Cortex_Ventral_AttentionA	1	
Ctx_i6_8_R	688	44	14	56	0,006490326288	Cortex_Fronto_ParietalB	1	
Ctx_VIP_L	608	-25	-67	66	0,005526341166	Cortex_Dorsal_AttentionA	1	
Ctx_SFL_R	1840	4	-3	74	0,006501335971	Cortex_Default_ModeB	1	

Multiple regions	4496	2	-37	-57	-0,01162206387	Cerebellum	7	spinal_trigeminal_R; spinal_trigeminal_L; dmnx_nts_R; dmnx_nts_L; Bstem_Med_R; Bstem_Med_L
Cblm_IX_L	1920	-1	-51	-37	-0,009644212243	Cerebellum	3	Cblm_Fastigial_L; Cblm_Vermis_X
Ctx_V1_R	17400	4	-77	-3	-0,02556308063	Cortex_Visual_Peripheral	1	
Cblm_CrusI_L	2448	-43	-69	-19	-0,01449357214	Cerebellum	2	Ctx_FFC_L
Cblm_CrusI_R	528	50	-63	-21	-0,007847099895	Cerebellum	1	
Ctx_V4_R	976	40	-83	-13	-0,01096754839	Cortex_Visual_Central	1	
Ctx_PIT_L	64	-49	-79	-15	-0,005808014346	Cortex_Visual_Central	1	
Ctx_V1_L	6296	-25	-95	-1	-0,01056513562	Cortex_Visual_Peripheral	2	Ctx_LO2_L
Ctx_V2_R	4224	24	-97	4	-0,01035544637	Cortex_Visual_Central	1	
Ctx_V3CD_L	320	-37	-89	18	-0,00553817815	Cortex_Visual_Central	1	
Ctx_V3A_R	1056	26	-89	24	-0,0107095185	Cortex_Visual_Peripheral	1	
Ctx_V3_L	64	-5	-99	22	-0,005083894901	Cortex_Visual_Central	1	
Ctx_IFJa_L	688	-41	18	30	-0,006057748102	Cortex_Fronto_ParietalA	1	
Ctx_V7_L	200	-27	-91	34	-0,005693968444	Cortex_Visual_Central	1	
Ctx_8BM_L	312	-3	36	38	-0,005389055494	Cortex_Fronto_ParietalB	1	

Regions from the CANlab 2018 combined atlas (https://sites.google.com/dartmouth.edu/canlab-brainpatterns/brain-atlases-and-parcellations/2018-combined-atlas), including the cortical atlas from (Glasser et al, Nature 2016), brainstem areas from (Shen et al, 2013), and the cerebellar atlas from (Diedrichsen et al, NeuroImage 2009). TG, area TG; pbn, parabrachial nucleus; IC, inferior colliculus; PeEC, perirhinal ectorhinal cortex; FFC, fusiform face complex; PoI, posterior insula; PIT, posterior inferotemporal; TA, area TA; FST, area FST; A4, auditory 4 complex; LO, lateral occipital area; Haben, habenula; PBelt, ParaBelt complex; PG, area PG; FEF, frontal eye field; VIP, ventral intraparietal complex; SFL, superior frontal language area; dmnx_nts, dorsal motor nucleus of the vagus/nucleus of the solitary tract; IFJ, area IFJ
